# Protocol for a scoping review of traditional medicine research methods, methodologies, frameworks and strategies

**DOI:** 10.3389/fmed.2024.1409392

**Published:** 2024-07-10

**Authors:** Nadine Ijaz, Jennifer Hunter, Suzanne Grant, Kate Templeman

**Affiliations:** ^1^Department of Law and Legal Studies, Faculty of Public Affairs, Carleton University, Ottawa, ON, Canada; ^2^Health Research Group, Sydney, NSW, Australia; ^3^Faculty of Medicine and Health, The University of Sydney, Sydney, NSW, Australia; ^4^NICM Health Research Institute, Western Sydney University, Penrith, NSW, Australia; ^5^Chris O'Brien Lifehouse, Sydney, NSW, Australia

**Keywords:** traditional medicine, complementary therapies, research methods, research paradigms, scoping review

## Abstract

**Background:**

The World Health Organization (WHO) has called for the evidence-informed integration of traditional medicine (TM) into health systems. Research rigor requires a good “fit” between research designs and what is being studied. The expectation that TM research fully adheres to biomedical evidentiary norms potentially creates tensions, as TM paradigms have their own distinct features. A scoping review will be conducted to describe and characterize the research approaches used in TM and their paradigmatic alignment with the TM being studied.

**Methods:**

This scoping review protocol was informed by Joanna Briggs Institute (JBI) methods. This protocol outlines an *a priori* conceptual framework, provisionally termed “paradigmatic alignment.” The review will include all populations, TM types, research approaches (i.e., methods, methodologies, frameworks, strategies), cultural contexts, and health care settings. Up to 38 English and non-English language databases will be searched sequentially for both published and gray literature until reaching data saturation across relevant concepts and contexts. Analysis will begin deductively, using a pre-piloted data extraction template to describe the TM research approaches. A basic qualitative content analysis of a sample of evidence sources will explore how research approaches are applied or modified to align with the TM therapeutic paradigm, and the manner in which they co-exist, contrast, complement or align with established biomedical research approaches. The findings will be narrated and summarized in charting tables and figures. The review will be reported according to the PRISMA scoping review extension. Consultative engagement with knowledge users across all review stages is planned.

**Discussion:**

Aligned with the principle of Two-Eyed Seeing (*Etuaptmumk*), wherein Indigenous/traditional and biomedical knowledges may equitably co-exist, this review promises to advance scholarly insights of critical value in an increasingly pluralistic, globalized world.

**Clinical trial registration**: https://clinicaltrials.gov/, identifier INPLASY2023110071.

## 1 Introduction

In 2020, the World Health Organization (WHO) announced the establishment of a new global center for traditional medicine (TM) in Jamnagar, India, to further advance the WHO’s long-standing call for governments worldwide to appropriately integrate TM into national health systems (1). The *WHO Traditional Medicine Strategy: 2014–2023* emphasizes that TM-related health system advancements “must be supported by evidence,” to foster delivery of safe, effective and accessible healthcare (2). However, surveys of WHO Member States consistently identify a “lack of research data” as their primary challenge in this regard (3). To support development of a research agenda for the new global TM center, the WHO has commissioned a series of evidence reviews. The scoping review protocol presented here pertains to one such commissioned study.

In the 2018 *Declaration of Astana,* the WHO explicitly called for the concurrent application of both biomedical and TM knowledges, and their associated practices, in advancing universal access to primary health care worldwide (4). There is strong academic and political pressure for TM to align with the biomedical evidence-based approaches that are widely applied in other areas of health and medicine. However, research methods must also be appropriate to what is being studied. TM takes many forms and is often underpinned by paradigms that differ in keyways from biomedicine (5). Tensions may thus arise in this context, as dominant biomedical research paradigms are not always optimally suited to studying TM approaches.

There remains an active global debate as to what types of research approaches may be most appropriate for the study of TM systems and their affiliated practices. This debate, in part, reflects the paradigmatic differences between TM and biomedicine (and its widely accepted research approaches). Since all research approaches—including standard biomedical methodologies and methods—are underpinned by their own paradigmatic features (6, 7), the question arises as to what types of rigorous research approaches may be aligned with TM paradigms. Indeed, the paradigmatic tenets of TM systems differ considerably from those underpinning dominant biomedical paradigms. Biomedicine is a therapeutic system historically structured around a worldview of “scientific materialism” that mechanistically reduces living systems to their constituent physical parts (8). Over the last 70 years, the biomedical paradigm has faced transformations from within, including the rise of a “biopsychosocial paradigm” (9), that includes social and psychological factors alongside biological considerations (10). In contrast, TM systems, while diverse, tend to be paradigmatically based in worldviews of “holism” (i.e., the whole is greater than the sum of its parts) (11), “vitalism” (i.e., there is a “vital” operating principle that distinguishes living organisms from other parts of the material world) (8), and/or “eco-centrism” (i.e., which situates the planet/nature rather than humans as the central conceptual element, drawing links between them) (11, 12).

TM researchers (13–15), like Indigenous scholars (16, 17), have critiqued the limitations of many mainstream health research norms, reporting methodological challenges with applying many standard biomedical research approaches (13). Some such critiques and challenges are similar to those raised and faced by biomedical researchers (18, 19). For example, in the field of psychotherapy (20), it has proven difficult to establish credible placebo/sham controls for many TM therapies (e.g., acupuncture, energy medicine) (21). Further—like some psychotherapeutic and psychosocial interventions in biomedicine—blinding within TM clinical trials is not possible when conscious awareness and/or learning is a component of the intervention itself (e.g., meditation, yoga, tai chi, expressive therapies). Notably, Cochrane reviewers have judged some psychosocial interventions (e.g., Alcoholics Anonymous, music therapy)—wherein active, participant engagement is (as in many TM therapies) a central therapeutic element—to be at low risk of performance bias, despite lack of blinding of study participants and personnel (22, 23). On the whole, a shift away from reductionist conceptualizations of health and disease has been evident in the biomedical research world in recent years, with a greater emphasis on biopsychosocial models, person-centered care, complexity science, and systems-based thinking (18). Part of this shift has included the development of research and evidence synthesis approaches that more rigorously evaluate health-related interventions—including TM approaches—with a high degree of intervention complexity (24). Further, considerations and strategies raised with respect to the cultural appropriateness of biomedical research approaches in TM contexts [e.g., Indigenous TM (25)] echo those discussed in biomedical contexts (e.g., “cultural psychiatry”) (26).

Despite these advances, many TM-specific challenges persist with respect to the alignment of biomedical research approaches with TM paradigms. For instance, the application of randomized controlled trial (RCT) study designs is challenging when evaluating interventions individualized according to TM-specific diagnostic frameworks, which are often strongly distinct from biomedical diagnostic categories; for example, in Chinese medicine, “liver qi stagnation” or “kidney yin deficiency” are common diagnostic categories (13). Related challenges are especially pronounced when a TM intervention is multicomponent (e.g., herbal formulations with multiple ingredients) and/or multimodal (e.g., acupuncture with herbal medicine)—and when treatments for a single patient may be modified over the course of a therapeutic process wherein the bodily conditions are understood to be changing (13). Other challenges arise from the dominant biomedical approach to pharmacological research, as this is based on a biomedical construct of a “single active ingredient.” However, the “one-disease one-target one-drug” notion is strongly at odds with many TM conceptual models for herbal medicines, wherein “whole plants” and plant combinations are understood to have various synergistic, additive and antagonistic effects on multiple targets with multiple clinical indications (27). Furthermore, in some TM systems, medicinal plants have spiritual significance and their therapeutic activity is understood in this light (5). Adding to this complexity, “relational” Indigenous knowledges “passed down through oral tradition,” as well as “Indigenous sources of knowledge, such as dreams, visions, or spirit” are often poorly considered or devalued within conventional biomedical research (28).

Research about TM—which in many settings carries both clinical and cultural importance—also raises complex sociopolitical and economic questions. For example, the “bioprospecting” approach that underpins much phytopharmacological research may, as the WHO and other United Nations agencies have observed, lead to large profits for pharmaceutical sellers, without concomitant recognition of Indigenous knowledge contributions, or benefit sharing with Indigenous communities (29). Further, it cannot be ignored that TM research—like TM’s integration into health systems—occurs within a sociopolitical context of biomedical dominance (5, 30), as well as inequitable conditions of resource distribution and knowledge production (5).

Over the last 20 years, there has been an exponential growth in TM-related research and evidence synthesis (31–33). Like biomedicine, the field of TM research crosses multiple disciplinary domains, including basic science, pre-clinical, clinical, health services, economic, policy, social science, ethnomedicine and implementation research. The methods and methodologies used range from quantitative to qualitative and mixed methods, Indigenous research designs, and machine learning and omics. Paradigmatic considerations, along with questions of Indigenous cultural and intellectual property, may arise across each of these domains, all of which shape the field’s broader, policy relevant evidentiary landscape. To date, however, systematic literature reviews that examine how paradigmatic considerations have been addressed in TM research have been lacking.

Multiple bodies of prior scholarship address the methodological issues to be investigated in this Review. Scholars in the TM field have critiqued the challenges posed by biomedical evidentiary hierarchies, proposing TM-relevant reconceptualizations (14, 15, 34, 35). Similar proposals have been advanced by biomedical researchers concerned with the limitations of dominant research norms (18, 19). Indigenous scholars have advanced a range of paradigmatically-aligned Indigenous research methodologies and methods, including decolonizing approaches, both within and beyond the health research field (16, 17). A previous systematic review by Saini explores areas of methodological “compatibility and convergence” between “[W]estern and [A]boriginal research designs” (36). In addition, TM scholars have developed several guidelines for the conduct and reporting of TM clinical research (37–42). Ijaz et al. scoping review of “whole systems research methods” details multiple adaptations to biomedical clinical research methods used by TM researchers (13). Some TM researchers have applied logic models and program theory to evaluate complex outcomes such as health behavior (43) and global functioning, as well as healthcare services that are integrating TM and biomedicine (44, 45). There are also examples of paradigmatically-aligned clinical practice guidelines for various conditions (46). Scholars seeking research approaches that align with TM therapeutic systems are furthermore adopting research approaches designed to evaluate complex interventions. Examples include, complexity science (47, 48) (which is the interdisciplinarity study of complex adaptive systems (49)) and systems science approaches (50), such as network pharmacology (51, 52) that have “shifted the paradigm from a “one-target, one-drug” mode to a network-target, multiple-component-therapeutics’ mode” (52). Likewise, TM-focused social scientists have demonstrated the importance of addressing paradigmatic considerations within TM policy processes (53, 54).

Notwithstanding these examples of TM research, a systematic review of the literature that scopes and summarizes the characteristics of TM research approaches, with reference to questions of paradigmatic alignment, is yet to be conducted.

The scoping review whose protocol is presented in this work therefore aims to: (a) summarize the range of research approaches (i.e., methods, methodologies, frameworks and strategies) currently used (or proposed for use) in the study of TM; and (b) describe the “paradigmatic alignment” of these research approaches with the TM being studied. Scoping reviews are well suited to answering “big picture” exploratory questions such as these, which do not seek to evaluate TM nor exhaustively map all TM research (55–57). As per JBI guidance, an *a priori* conceptual framework, provisionally named “paradigmatic alignment,” will steer the review process. The following sections contextualize the review methods by elaborating key definitions and presenting the project’s conceptual framework.

## 2 Methods

An abridged version of this protocol was registered on 17 November 2023 on the International Platform of Registered Systematic Review and Meta-analysis Protocols (INPLASY2023110071) (58). The methodology for this scoping review is informed by JBI guidelines (56), and will be reported according to the Preferred Reporting Items for Systematic Review and Meta-analysis Scoping Review extension (PRISMA-ScR) (59). This protocol is reported according to a modified PRISMA-P reporting checklist (Supplementary File S1) (60).

### 2.1 Research question

The primary, two-part question driving this scoping review is:

- What are the types and characteristics of the research approaches (i.e., methods, methodologies, frameworks and strategies) applied in, or recommended for application in, TM research; and
- How do these TM research approaches and their affiliated research paradigms align with the therapeutic paradigm(s) of the TM being studied (i.e., paradigmatic alignment) (Table 1)?

**Table 1:** Paradigms and worldviews.

Research paradigm	Therapeutic paradigm	Indigenous worldview
Ontology	Ontology	Ways of being
Epistemology	Epistemology	Ways of knowing
Methodology	Clinical practice	Ways of doing

Three related sub-questions are:

1. Where (e.g., regions, countries), by whom (e.g., cultural groups, communities/patients, practitioners, professions, governments, academic institutions) and why (e.g., rationale, purpose) are these TM research approaches being applied or recommended?
2. How have TM research approaches been applied (and/or modified) to suit different TM types, knowledges and practices, and regional, disciplinary or cultural conditions?
3. How do paradigmatically-aligned TM research approaches co-exist, contrast, complement or align with established biomedical research approaches?

### 2.2 Definitions and concepts

#### 2.2.1 Traditional medicine

The WHO defines traditional medicine (TM) as

*the sum total of the knowledge, skill, and practices based on the theories, beliefs, and experiences [I]ndigenous to different cultures, whether explicable or not, used in the maintenance of health as well as in the prevention, diagnosis, improvement or treatment of physical and mental illness* (2).

Further, as WHO observes, some TM approaches may be used outside of their geographies and cultures of origin and are sometimes categorized as “complementary medicine” in such contexts.

While the WHO’s definition of TM will provide a basis for the review, that definition is primarily theoretical (i.e., broadly characterizing the TM construct) rather than operational (i.e., specifying what is included within the construct). Ijaz has elsewhere advanced an operational typology theoretically based on the WHO’s definition (61). Two key features of the typology make it a suitable basis to inform the review.

First, it is broadly inclusive of a wide range of Indigenous, ethnomedical and otherwise-unconventional therapeutic approaches that are widely used across the globe. This inclusive approach is consistent with the WHO’s own indications that it intends the TM construct, in the context of its new global TM center, to include a wide range of “traditional, complementary, and integrative medicine” approaches (1).

Second, the typology draws close attention to knowledge paradigms as a core differentiating factor between TM approaches, whether codified (e.g., Ayurveda, Chinese medicine, homeopathy) or non-codified (e.g., many local Indigenous healing approaches and remedies in the self-care domain). It also acknowledges the overlap between therapeutic approaches that are rooted in TM paradigms, yet strongly incorporate biomedical paradigmatic perspectives (e.g., anthroposophy, chiropractic, naturopathy, osteopathy), and other complementary therapeutic approaches that have developed alongside biomedicine when practiced within the context of a biomedical paradigm (e.g., dry needling, dietary supplements used in isolation to treat a symptom or disease).

#### 2.2.2 Research approaches and domains

In this review, the term *research approaches* will be used when referring to any aspect of the research process (i.e., methods, methodologies, frameworks, and strategies). Research *methods* refers to the specific techniques for collecting, analyzing and reporting research data. The research *methodologies* are the guiding principles and study design that inform the selection and application of the research methods. The theoretical context, rationale and/or guidelines for conducting research may be outlined in a research *framework*. Research *strategies* are the structured plans for achieving the research goals, whether for a single project or an entire field, and may encompass the overall study design(s), task sequencing, and resource allocation.

WHO Member States consistently signal the need for more TM research data to inform regulatory decisions (3). Along with clinical research evaluating TM effectiveness and safety, other evidence (information) is also required when making clinical decisions, providing public health guidance, regulating therapies and practitioners, and delivering health services. For instance, in addition to evidence about the clinical effectiveness and safety of a TM intervention, evidence about modifying factors (e.g., patient/population use; stakeholders’ preferences and values; direct and indirect costs; availability, quality control, and regulatory consideration; and the benefits and risks of alternative option, including doing nothing) may warrant recommending or implementing a TM intervention despite low certainty in the clinical evidence and vice versa (62, 63). Another example is the United States of America Food and Drug Administration endorsed framework for evaluating the safety of dietary supplements considers evidence from historical (traditional) use, *in vitro* and animal studies, clinical trials, epidemiological studies, and post-market surveillance (64).

Therefore, the scoping review will examine the research approaches that are applied across a wide range of disciplinary *research domains* including basic science and preclinical research, clinical research, health services research, health economics, health technology assessments, implementation science, social sciences, ethnomedicine, policy research, and Indigenous methodologies [e.g., storytelling, yarning, talking circles, and other culture-specific methods (65)].

#### 2.2.3 Conceptual framework: paradigmatic alignment

Conceptual frameworks can be a “critical element in effectively focusing [a] review and designing the methods to respond to the knowledge question” (66). In line with JBI guidance, an *a priori* conceptual framework will inform all stages of the scoping review’s conduct, from sub-question development to search strategy and data analysis (56). This conceptual framework, provisionally termed “paradigmatic alignment,” refers to the ways in which a research approach (and its paradigmatic underpinnings) align with the paradigmatic features of the TM therapeutic approach being studied. The principles of Two-Eyed Seeing (*Etuaptmumk*) (67) and a TM-specific concept of “model validity” (13) were used to inform the theoretical underpinnings of this framework and it operationalization.

##### 2.2.3.1 Research and therapeutic paradigms

The term *paradigm* is typically used when referring to “the entire constellation of beliefs, values, techniques, and so on shared by the members of a given community” (68). Based on the work of research methodologists, who have extensively elaborated on the concept of a “research paradigm,” for the purpose of this scoping review, paradigms will be understood to include three central elements: *ontology* (understandings about the nature of reality); *epistemology* (how knowledge is acquired, constructed and justified); and *practice* (e.g., methods, methodologies and techniques) (6, 7). Another reason for using this three-part construct is its alignment with the work of Indigenous scholars, who often refer to “*ways of being, knowing and doing*” when describing Indigenous “worldviews” (Table 1) (69, 70).

###### 2.2.3.1.1 Research paradigms

Research methodologists have expounded upon “research paradigms” (such as positivism, post-positivism, realism, constructivism, interpretivism, Indigenous research paradigms) and their application according to the research purpose and context (6, 7). The research paradigm directs the choice of the study design, data collection, analysis, and interpretation. While a specific research method or methodology may not exclusively belong to a single research paradigm, they will often align with certain research paradigms. For example, quantitative methods typically align with the positivist paradigm, while qualitative methods lend themselves to research that draws on constructivism or interpretivism (7). Notably, it is widely acknowledged that positivist and post-positivist research paradigms (along with associated quantitative methodologies like RCTs) hold sociopolitical privilege over other research paradigms and approaches (6, 7, 18). Further, it will be taken as axiomatic that every research approach has its own ontological and epistemic basis regardless of whether this is explicitly recognized by academic scholars.

###### 2.2.3.1.2 Therapeutic paradigms

Although the term “therapeutic paradigm” is commonly used, it has not been extensively theorized. Notwithstanding, educationalists from various healthcare disciplines have discussed how therapeutic and disciplinary paradigms, along with their related clinical practices, are influenced by the ontologies and epistemologies of both the individual practitioner and their professional bodies (71, 72).

In a comparative analysis of four complex medical systems—contemporary biomedicine, homeopathy, traditional Chinese medicine, and Ayurveda—Brazilian scholar Madel Luz proposed the “medical rationalities” framework (73). Luz observed that all four systems exhibited the characteristics of a complex medical system, as they each have their own distinct cosmology (a construct similar to ontology), elements in which that cosmology is expressed as knowledge, or epistemology—including medical doctrine (that defines and guides foundational concepts for health, disease, etiology, and clinical practice), morphology (that describes that body’s micro and macro structures and anatomy), and physiology (that explains the body’s functional and regulatory systems) –and, areas in which epistemology and practice intersect—that is, diagnostic and therapeutic systems. In this sense, Luz’s model of a “complex medical system” may be provisionally understood as similar to the concept of a “therapeutic paradigm.”

Like biomedical paradigms, TM paradigms are not static and continue to evolve and shift (74, 75). In contrast however, biomedical therapeutic paradigms typically wield more political influence globally than contemporary TM therapeutic paradigms (5).

##### 2.2.3.2 Two-eyed seeing (*Etuaptmumk*)

Two-Eyed Seeing, or *Etuaptmumk* (pronounced: eh-doo-ahp-duh-mumk), is a principle advanced by Mi’kmaq Indigenous scholars Marshall and Marshall wherein Indigenous/traditional knowledges and biomedical knowledges may equitably co-exist (67).

As the authors explain, Two-Eyed Seeing (*Etuaptmumk*):

*refers to learning to see from one eye with the strengths of (or best in) Indigenous knowledges and ways of knowing, and learning to see from the other eye with the strengths of (or best in) Western knowledges and ways of knowing… [and] using both of these eyes together for the benefit of all. We need to utilize Two-Eyed Seeing to determine the benefits both in the modern medical science knowledges and in our Indigenous knowledges … Two-Eyed Seeing encourages that we draw upon both new technologies and traditional practices to lead to better health outcomes for everyone* (67).

This principle has been widely engaged in health research addressing traditional/Indigenous knowledges and ways of knowing, and adopted by government agencies (such as the Canadian Institutes of Health Research) (76). Constituting “Indigenous and Western *knowledge systems* as whole and, distinct in and of themselves, Two-Eyed Seeing holds that each knowledge system can only offer a partial understanding of the world … [and recognises that] no single worldview offers everything” (76). In the context of this scoping review, the central importance of Two-Eyed seeing as a guiding principle is that it explicitly recognizes—within a context of biomedical dominance—the concurrent and fundamentally equal value of distinct forms of knowledge, even though each will necessarily contribute to the domains of research, policy and clinical practice in its own way. In this sense, the principle of Two-Eyed Seeing (*Etuaptmumk*) is closely aligned with the principles of epistemic pluralism and epistemic equity, as explained below.

###### 2.2.3.2.1 Epistemic pluralism and epistemic equity

Epistemic pluralism recognizes that multiple epistemologies (be they various biomedical or TM knowledges) may co-exist despite unequal sociopolitical power dynamics (77). It recognizes that the dominant epistemologies may become so deeply embedded within sociopolitical structures and mass culture that their underlying assumptions appear “neutral” or “universal” despite being culturally and historically situated (78). In this context, the concept of “epistemic equity” or “epistemic justice” holds that diverse epistemologies should be appropriately and fairly valued according to their unique contributions (77). Such an equitable co-existence of diverse epistemologies has also been termed “an ecology of knowledges” by decolonial scholars (79). These principles are closely aligned with the WHO’s call for the appropriate integration of TM within biomedically dominant national health systems worldwide (2, 4).

##### 2.2.3.3 Model validity

Model validity is a term that holds multiple meanings across different research communities. However, for the purpose of this review, the TM-specific use of the term will be engaged. Originally referred to as “model fit validity” by Cassidy (80), TM clinical researchers have distinctly used the term “model validity” to denote a principle that emphasizes the importance of aligning clinical research paradigms with the TM being studied (13, 81–84). With reference to biomedical contexts, where there exist commonly held paradigmatic assumptions about etiology, diagnosis, pathophysiology, and outcomes, Jonas and colleagues explain that there is often no “need to evaluate whether research methods have violated these basic assumptions” (81). However, in TM research, whose paradigmatic assumptions diverge from those biomedical, the concept of model validity gains in importance.

Much TM scholastic work has involved the development and application of specific criteria for critically appraising the model validity of TM clinical research (81–84). However, leading on from this, in a scoping review of TM whole systems research, Ijaz and colleagues used the TM-specific construct of “model validity” in their conceptual framework that “represents a commitment to actively preserving these [TM] paradigms and practices in their own right” (13). That conceptual framework aligns closely with the WHO’s commitment to “protect[ing] traditional knowledge,” articulated in the TM Strategy 2014–2023 (2).

Ijaz and colleagues proposed three inter-related subcategories of the model validity construct—paradigm compatibility, paradigm consistency, and paradigm specificity—with reference to TM clinical research (13). In this scoping review, the model validity construct and its three subcategories will be analytically applied to a wide range of research approaches and domains (including, but not limited to, clinical research) and, thus, require further theoretical elaboration and development. As explained below, this includes a minor adjustment to Ijaz et al. concept of “paradigm consistency,” renaming it to “paradigm adapted,” with the addition of a fourth subcategory of “paradigm pluralistic.”

*Paradigm specific* research will refer to research approaches originating in a specific (therapeutic or research) paradigm that are best suited for the conduct of studies within that same paradigm. For example, placebo controlled, RCTs were developed by biomedical researchers to evaluate the efficacy of single drugs when used for a single diagnosis and quantifiably-measured outcomes. Similarly, there are traditional and Indigenous research approaches that were designed to study their own practices. For instance, there are numerous examples of culturally specific Indigenous research methods, such as talking circles, storytelling and “yarning” (65). Notably, culturally specific methods that originated in one Indigenous group may be paradigm specific and, therefore, not translatable to other Indigenous groups due to differences in their cultural practices, paradigms, and worldviews (65).

*Paradigm compatible* research, by contrast, is a research approach that originates in one paradigm yet may also be suitable for studying a different therapeutic paradigm. For instance, a double-blind, placebo-controlled RCT may be used in a model valid manner to evaluate TM interventions where: (a) an inert placebo can be created; (b) a TM therapeutic approach is traditionally used in isolation to treat a singular “generic” symptom; and/or (c) application of a TM therapeutic approach does not require specialized TM diagnosis or therapeutic individualization (e.g., a standardized herbal formula, supplement, or other TM drug for headache, cough, soft-tissue bruising, menopausal hot flashes, sleep quality etc.).

Pragmatic trial designs that use various randomization and recruitment methods, and often include real-world data to evaluate complex interventions in the setting where they are intended to be delivered, are another example of a research method originating in biomedical science that have been appropriately used to evaluate complex, individualized TM interventions in a model valid way (13). Similarly, researchers seeking to investigate complex TM herbal formulations (and their synergistic effects) use network pharmacology methods, developed in biomedical contexts to investigate synergistic drug combinations (85–87).

*Paradigm adapted* research approaches are those developed within one knowledge system adapted or modified to better fit another paradigmatic context. Examples include the adaption of a biomedical clinical trial method to include TM diagnoses in the inclusion criteria and/or for individualized treatment protocols (13), or community-based participatory research methods that are modified to incorporate “yarning” or “storytelling” (which are culturally-situated methods based in Indigenous oral traditions) (65). The TM extensions to CONSORT and PRISMA reporting guidelines are another example of such paradigmatic adaptations to established research approaches.

Finally, a fourth subcategory, termed *paradigm pluralistic,* will be provisionally added to better operationalize the model validity construct within this scoping review. Paradigm pluralism represents areas of interweaving between paradigms where two or more research approaches from different paradigms are used side-by-side. Examples include Indigenous-led co-research methods that combine ethnobotanical and preclinical research (88), clinical practice guidelines that report the confidence in the TM knowledge along with the certainty/quality of empirical research (89), and regulatory guidance for industry on how to synthesize both TM knowledge and empirical research when making health claims for regulated TM products (90).

Overall, the principle of Two-Eyed Seeing (*Etuaptmumk*) (67) is expressed across all four model validity subcategories, illustrating different ways in which biomedical and TM knowledges have the potential to fruitfully, dynamically and respectfully co-exist in research-related contexts.

### 2.3 Eligibility criteria

The inclusion criteria will follow the JBI scoping review framework of concept, context, and types of evidence sources (56).

In summary, the evidence sources to be included in this review will:

1. Describe or summarize the research approaches used to study TM;
2. Propose, recommend, or provide guidance for TM research;
3. Critique or articulate a rationale for developing, implementing, or applying TM research approaches; and/or
4. Provide an example of paradigmatic alignment between a specific research approach and the TM being studied.

#### 2.3.1 Concepts

The concepts of interest that will inform the inclusion and exclusion criteria are: (1) traditional medicine, (2) research approaches, and (3) the review’s conceptual framework of paradigmatic alignment that encompasses the concepts of research paradigms, therapeutic paradigms, Two-Eyed Seeing (*Etuaptmumk*) (67) and the TM-specific use of the “model validity” concept (13).

Any form of TM that aligns with the WHO’s definition will be included (2, 61). Medicines with natural ingredients or derivatives when used in a biomedical setting (e.g., vitamin K for neonates, digoxin for cardiac conditions, vincristine for cancer treatment) will be excluded (91). As will biomedical healthcare approaches and related disciplines (e.g., psychiatry/psychology, physical/physiotherapy, exercise physiology, rehabilitation, occupational therapy, dietetics, lifestyle medicine, public health interventions etc.). The exception are instances when biomedical approaches are used alongside or integrated with TM (91).

The included evidence sources will describe or summarize TM research methods, methodologies, frameworks or strategies (i.e., research approaches). Sources that only discuss TM therapeutic paradigms (along with their associated knowledges and practices) will be excluded.

Based on the primary review question, technically, all documents reporting TM research could be included. Therefore, for pragmatic reasons, the following will be excluded and will not be actively searched for:

1. Primary studies of TM, except those where it is not possible to identify a literature review that summarizes the relevant methods or methodologies, or when the primary study is deemed to be an exemplar.
2. Secondary studies, review articles, etc. that do not describe, discuss, or critique a TM research approach.
3. Secondary studies, review articles, etc. that only evaluate or discuss TM efficacy, effectiveness, safety, mechanisms of action, or the risk of bias / quality of primary TM studies. Instead, we will include overviews/umbrella reviews, mapping reviews, scoping reviews etc. that summarize review methods and methodologies. The exception will be when paradigmatically aligned synthesis methods or critical appraisal tools are used, or reviews that report a wide range of study designs.
4. Clinical practice guidelines and related consensus statements / Delphi studies for diagnosis or treatment.
5. TM monographs and related evidence sources.

#### 2.3.2 Context

The contexts that will inform the inclusion and exclusion criteria for the scoping review are as follows.

All research domains (e.g., basic science, preclinical, clinical, health services, economic analyses, health technology assessment, implementation science, social sciences, ethnomedicine, and policy research) will be included. Indigenous methodologies such as storytelling, yarning, talking circles, and other culture-specific methods (65), that involve research “of” Indigenous peoples will only be included if the context is TM, either as a standalone concept or when integrated with biomedicine and/or mainstream health services.

All geographical locations, countries, regions, cultural contexts, and health care settings (e.g., community, self-care, primary care, secondary care, integrative or traditional health care, clinical or non-clinical settings) will be included. However, research or activities that do not directly pertain to human health or health care will be excluded, as will research for other purposes such as veterinary, agriculture, or the environment.

#### 2.3.3 Types of evidence sources

The types of evidence sources to be included are broad, including primary and secondary research articles and other types of articles published in peer reviewed journals (e.g., viewpoint and review articles, guidelines for the conduct or reporting of TM research), gray literature (e.g., research theses, guidelines, white papers, reports, and policy documents), and books and book chapters. Inclusion of gray literature is important in the present context due to known barriers faced by TM stakeholders regarding participation within biomedically-dominant scholarly contexts (34, 92–94). Information published on websites, letters to editors, conference proceedings/abstracts, magazine and news articles, and other evidence sources will be excluded unless this is the only example identified in the search for a particular research approach or a key example of paradigmatic alignment. There are no date nor language restrictions, and all efforts will be made to translate potentially relevant evidence sources.

### 2.4 Search strategy

Free-text and standardized search terms will reflect general overarching TM terms, Indigenous health care approaches, TM whole systems, overarching terms for botanicals and nutraceuticals, and terms for individual TM modalities and therapies. The selection of terms were informed by search terms identified by Ng et al. operational definition of complementary, alternative and integrative medicine for literature reviews (95). The final selection of terms aimed to optimize a balance between sensitivity and specificity by focusing on the TMs terms that contrast biomedical therapeutic paradigms as outlined in a TM typology proposed by Ijaz (61) and an operational definition of Integrative Medicine for primary care research proposed by Hunter et al. (91). TM terms will be combined with terms for research approaches, methodological approaches for evaluating complex phenomena, and other review concepts (e.g., epistemology, paradigm, model validity, fit-for-purpose). Search term syntax, fields and languages will be tailored according to the database that is searched.

**Figure 1:**
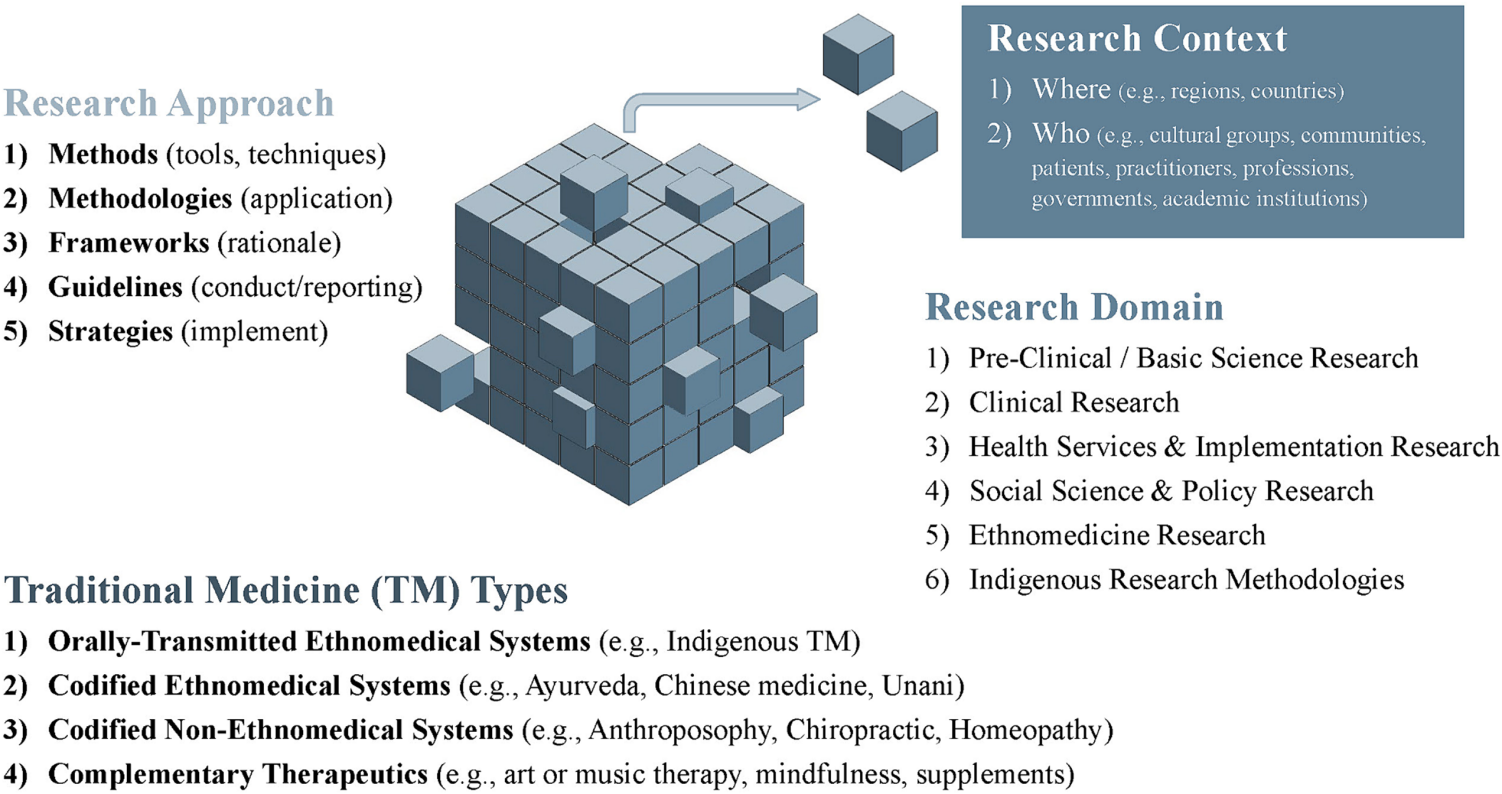
Scoping review matrix.

To account for the exploratory nature of the scoping review question and sub-questions, database searching will be staggered and continue concurrently with analysis until data saturation is reached (i.e., no new information is identified through further literature searching). This approach draws on qualitative research methodology where iterative techniques, such as the constant comparative method, are applied when determining data saturation (96). While all TM, research domains and research approaches will be included, it is impractical to search for, and include every published example. Therefore, data saturation will focus on sampling the overarching categories outlined in scoping review matrix (Figure 1). These are: (a) the TM research approaches (methodologies, methods, strategies and frameworks); (b) TM research domains (basic science/preclinical research, clinical/health services, and social science/ethnomedicine); (c) different types of TM systems and practices (non-codified Indigenous, codified whole systems, and other complementary therapeutic approaches including self-care); and (d) the various contexts (location, knowledge users) where these TM research approaches were applied, discussed or recommended. To align with the review’s focus on paradigmatic alignment, data saturation will emphasize TM therapeutic paradigms that sharply contrast biomedical paradigms.

The Category I databases listed in Table 1 will first be searched using the free-text and standardized search terms outlined in Supplementary File S2 that were designed for the English language. Notwithstanding this English-language bias, relevant articles/citations published in languages other than English will be included and the index terms will be tailored for the databases searched. When data saturation is not reached, additional focused searches for a key population, concept, and/or context will be conducted, including non-English languages (e.g., Arabic, Chinese, French, Hindi, Japanese, Korean, Spanish, Portuguese, Thai) with corresponding search strategies using one or more of the Category I or II databases (Table 2). Except for some secondary focused databases searches, databases will be searched from inception.

**Table 2:** Scoping review and mixed methods systematic review databases to be searched.

Category I	Category II
**EBSCOhost** Academic Search Complete Anthropology Plus CINAHL Complete Humanities Source Ultimate Psychology and Behavioral Sciences Collection Sociology Source Ultimate **Ovid** Allied and Complementary Medicine Database (AMED) EBM Reviews—Cochrane Methodology Register MEDLINE APA PsycInfo **Global Index Medicus (WHO databases)** African Index Medicus (AIM) Index Medicus for the Eastern Mediterranean Region (IMEMR) Index Medicus for the South-East Asia Region (IMSEAR) Latin American and Caribbean Health Sciences Literature (LILACS) Western Pacific Region Index Medicus (WPRO)	African Journals Online (AJOL) Applied Social Sciences Index and Abstracts (ASSIA) AMEDfind (AMED R&D Project Database) AYUSH Research portal China National Knowledge Infrastructure (CNKI) Chinese Scientific Journals Database (VIP) Core Outcome Measures in Effectiveness Trials (COMET) Database Digital Helpline for Ayurveda Research Articles (DHARA) eMarefa (Digital Arabic Database) Embase Enhancing the QUAlity and Transparency Of health Research (EQUATOR) Ethnic NewsWatch Google Scholar Indexing of Indian Medical Journals (INDMED) Japan Medical Abstracts Society (Ichushi-Web) Japan Science and Technology Information Aggregator J-STAGE Korean studies Information Service System (KISS) Ministry of health, labor and Welfare (MHLW) Grants system database (Japan) Scopus Shodh Ganga Thai Journals Online (ThaiJO) ThaiLIS/Thai Digital Collection (TDC) Wanfang Data WHO Institutional Repository for Information Sharing (IRIS) Web of Science (all databases)

Protocol-driven search strategies, will be used alongside iterative search methods, such as citation tracking (97), pearl growing/snowballing (97, 98), and CLUSTER searching (99). This strategy is recommended for systematic reviews of complex evidence (97–100). Other tools such as PubMed Miner (101) will be used to refine additional database search strategies. Searches will be supplemented by information and documents known to members of the Review Team, the WHO TM Evidence Task Force, and other knowledge users through the Review Team’s regional outreach strategy. Authors will only be contacted about key information that is needed to directly answer the review’s questions. All procedures will be documented for transparent reporting.

#### 2.4.1 Screening, selection, and data extraction

All identified citations from database searches that can be exported in RIS format will be collated and uploaded into EndNote 20 software (102) for automatic linking of available full texts and initial screening for duplicates. Covidence systematic review software (103) will then be used for a second screening of duplicates, that will be followed by title/abstract screening, full text screening, and preliminary data extraction of the included articles. For database searches where this strategy is not feasible, we will use alternate comparable methods.

Following calibration exercises, two reviewers will independently conduct title/abstract and full text screening. Inter-rater reliability will be checked and reported using the Covidence functions. Due to the broad nature of the research question, over 10,000 citations may need to be screened. If the inter-rater reliability is high after the first round of screening (e.g., percentage agreement >80%), rapid review methods may be employed for additional rounds wherein, following calibration exercises, single reviewers screen and a second reviewer screens the excluded citations (104). Disagreements between reviewers will be resolved via consensus.

Single reviewers will extract data according to a prespecified, piloted data extraction template in Covidence (Supplementary File S3—draft extraction form); a second reviewer will verify data extraction. The details of any modifications will be reported. Disagreements will be resolved via consensus. Electronic spreadsheets and Taguette software (105) will be used when extracting, sorting and categorizing data for the supplementary qualitative data analyses.

### 2.5 Quality appraisal

Quality appraisal (e.g., risk of bias) assessments will not be conducted for all included evidence sources. However, if the evidence source was not peer reviewed, then prior to using it to inform the in-depth, qualitative analysis, the JBI Critical Appraisal Tools will be used (106). Notably, the JBI suite includes appraisal tools for different types of textual evidence and all checklists have an overall appraisal recommendation to include, exclude, or seek further information. Single, senior reviewers will conduct the appraisals. The decision to include or exclude an evidence source will be verified by another senior reviewer. Disagreements will be resolved via consensus.

### 2.6 Analysis and evidence synthesis

The analysis will follow JBI guidance for scoping reviews (56, 107). To account for the substantial heterogeneity and diversity of data that is likely to result from the study’s broad, exploratory research questions and the multidimensional sampling frame, subsequent analysis and synthesis of findings, will proceed in stages. This will be informed by the review’s conceptual framework of paradigmatic alignment and related concepts previously outlined.

The first stage of the analysis will focus on describing the types and range of research approaches used to study the various forms of TM across diverse contexts. Data extracted in Covidence will be exported into electronic spreadsheets. Analysis will begin by summarizing the characteristics of the evidence sources (e.g., citation details, publication type and year, countries of authors, declarations) and the types of TM, research domains and research approaches that were applied, discussed or recommended. The scoping review matrix in Figure 1. illustrates the overarching categories that will be used for sorting and charting the data. Additional subcategories may be inductively introduced or developed over the course of the analytic process.

Data extraction in Covidence will include preliminary coding to flag potentially relevant evidence sources (Supplementary File S3—data extraction form) for further in-depth analysis. A basic qualitative content analysis (107) will then be conducted to explore the question of paradigmatic alignment and other related sub-questions. After data immersion, analysis will initially proceed deductively in relation to the review’s conceptual framework and the scoping review matrix (Figure 1). Reviewers may also flag emergent topics that were not addressed in the *a priori* sub-questions yet appear relevant to the review. Other qualitative methods, such as “following the thread” (108), the constant comparative method (96), and deductive and inductive thematic methods (109, 110) may, therefore, be indicated as the qualitative content analysis proceeds. After reaching consensus about further topics or sub-questions that warrant further exploration, literature searching and analysis will then continue until saturation is reached. All *post hoc* changes or modification to this protocol will be transparently reported.

Further, in seeking answers to a specific sub-question, or in characterizing a particular topic, reviewers will identify illustrative exemplars. To facilitate knowledge user engagement with review findings, some of these exemplars will be highlighted in the results. When selecting and reporting exemplars, we will aim toward regional and TM disciplinary inclusivity.

A narrative summary of the finding supported by relevant tables and figures will characterize the diverse range of research approaches employed in TM research. Draft charting tables are reported in Supplementary File S4.

### 2.7 Knowledge user engagement

Involving knowledge users (stakeholders) recognizes their perspectives, expertise, and experiences, ensuring research outcomes are relevant, applicable and useful for end-users. In line with JBI guidance on knowledge user engagement for scoping reviews (111), from the outset, a Review Team was formed, composed of two dozen TM research experts from all global regions (see: Acknowledgements). Review Team members are primarily established scholars in the TM field with training and expertise in a wide range of research methodologies (including biomedical research methods, sociology, implementation science, and Indigenous research methodologies) as well as clinician-scientists with dual biomedical-TM training.

Review team members were also selected with the following principles in mind: regional and contextual representation and representation across diverse research domains and TM types. Following WHO requirements, all Review Team members must formally disclose their varied engagements to manage potential conflicts of interest. The team members provide expert advice on all stages of the review. Membership may be expanded to fill key knowledge gaps. Review Team members will also support regional outreach through their networks. Additional stakeholder consultations that informed the development of this protocol included a roundtable discussion between TM experts appointed by the six WHO regional head offices at the 2023 WHO Traditional Medicine Global Summit, India (August 2023), and feedback from the TM Evidence Task Force of global experts appointed by the WHO to advise on the conduct of commissioned reviews.

## 3 Discussion

This protocol outlines plans for a scoping review that will describe the range of research approaches used to study various forms of TM and characterize these approaches with reference to the concept of “paradigmatic alignment.” Commissioned by the WHO, the review aims to support development of a policy-relevant global TM research agenda. At once, the review’s design seeks to advance the WHO’s articulated goal of TM’s evidence-informed integration within health systems globally, while explicitly recognizing the value of traditional and Indigenous therapeutic knowledges within a biomedically-dominant therapeutic landscape. The review findings will seek to highlight methodological trends and innovations in paradigmatically aligned TM research. As such, the work will reflect demands for healthcare to be informed by the best available evidence.

The conceptual framework proposed for this review aligns well with broader trends in the biomedical context of evidence-based medicine (EBM). “A New Framework for Causal Inference in the Health and Social Sciences” (112), often referred to as EBM+ (18, 113), calls for what is increasingly termed *evidential pluralism*. Proponents of this framework are challenging the dominant evidentiary hierarchy, “which favor[s] probabilistic evidence from clinical trials” (18). The importance of integrating other sources of evidence, such as mechanistic studies and a broader range of quantitative and qualitative evidence, is emphasized (18, 113, 114). Although non-biomedical therapeutic paradigms are not explicitly addressed, the underlying tenets of evidential pluralism echo the perspectives of TM scholars who have similarly critiqued the limitations of biomedicine’s evidentiary hierarchy (13–15, 35). Evidential pluralism also parallels the WHO’s recognition of both “scientific” (formal) and “tacit” (informal, stakeholder-informed) forms of evidence as contextually relevant to policy making (115). Critically, the planned scoping review extends biomedical critiques of evidentiary norms to recognize the potential contributions of TM therapeutic and research paradigms—that is, their ontologies, epistemologies and practices—to the broader world of health care.

The broad, exploratory nature of the research questions, coupled with the inclusion of a wide range of disciplinary approaches to research, aims to ensure that the breadth of TM research required for clinical decision-making and policy is described. Notwithstanding this strength, the decision will present substantial logistical challenges. There are thousands of potentially eligible evidence sources that are indexed across hundreds of databases in different languages. We considered limiting the search date parameters. However, key documents such as the WHO *General Guidelines for Methodologies on Research and Evaluation of Traditional Medicine* (116), published in 2000, would then be excluded. Instead, other compromises are planned. Evidence sources published in languages other than English will not be identified during the first round of database searches, unless they are indexed with the searched controlled vocabulary terms (e.g., MeSH, DeCS), the title or abstract is also indexed with an English-language translation, or they are already known to the review team. Additionally, instead of conducting an exhaustive literature review, we plan to limit our analysis to only describing and characterizing the range of research approaches. Literature searching will stop when data saturation is reached. Thus, the search strategy cannot accurately quantify the frequencies of different research approaches nor to identify gaps in their application according to TM type, health care setting or region.

Operationalizing the conceptual framework is another potential challenge with the execution of this protocol. Although a similar framework was previously used in a scoping review of whole systems clinical research (13) its broader applicability is yet to be ascertained and as such, may require further modifications as the analysis proceeds. As per scoping review guidance that cautions against reviewers synthesizing or critically appraising the findings (107, 117), theoretical, iterative and synthesis methods will not be used. The review will stop short of judging, critiquing or recommending specific research approaches. Notwithstanding, the findings of this review will lay the groundwork for further studies that are designed to explore and interrogate the complexities and challenges with conducting, synthesizing and implementing TM research across global contexts.

Engaging with knowledge users throughout all stages of a literature review is another design strength (111). However, due to practical constraints, most of the knowledge users involved in this scoping review are TM researchers, many of whom reflect either the authors’ or the WHO’s academic networks. Despite efforts to achieve diverse global and disciplinary representation, there are inevitable gaps. Some of our knowledge users have dual roles as healthcare practitioners or possess broader non-TM research, health service or policy experience. However, there will only be limited, informal engagement with other key stakeholders, such as patients, communities, and practitioners who are not researchers, health service providers, and policy-makers.

Finally, the limitations of the scoping review methodology must be noted. Like other “big picture” reviews (57), scoping reviews are not designed to answer specific questions, such as which TM research approaches are most suitable or should be recommended. Instead, by describing and cataloging the range of research methods, methodologies, frameworks and strategies that have been applied or recommended the findings will provide contextual information about their potential roles in conducting paradigmatically aligned research.

Even though the planned scoping review is focused on a defined field of interest—TM research—with reference to a conceptual focus of paradigmatic alignment, its design, conceptual underpinnings, and anticipated findings may have broader relevance. As previously noted, scholars, clinicians, and decision makers in other fields face similar challenges due to intervention or system complexity, or misalignment of their therapeutic paradigm with research norms. By recognizing the value of multiple approaches to knowledge and practice, the scoping review seeks to advance scholarly insights of critical value in an increasingly pluralistic and globalized world.

## Author contributions

NI: Conceptualization, Funding acquisition, Methodology, Project administration, Validation, Writing – original draft, Writing – review & editing. JH: Conceptualization, Funding acquisition, Methodology, Project administration, Validation, Writing – original draft, Writing – review & editing. SG: Validation, Writing – review & editing. KT: Project administration, Validation, Writing – review & editing.
