# Supplementary material for: Protocol for a scoping review of traditional medicine research methods, methodologies, frameworks and strategies
Source: Front Med (Lausanne). 2024 Jul 10;11:1409392. doi: 10.3389/fmed.2024.1409392 (PMC11267516; doi:10.3389/fmed.2024.1409392)
Supplement: Supplementary file 1 [file Data_Sheet_1.pdf]

frontiers

Supplementary Material

Protocol for an Evidence Review of Traditional Medicine Research Methods, Methodologies, Frameworks and Strategies.

Nadine Ijaz*†, Jennifer Hunter†, Suzanne Grant, Kate Templeman

* Correspondence: Nadine.Ijaz@Carleton.ca

†These authors contributed equally to this work and share first authorship

1 Reporting Checklist

Table 1. PRISMA-P (Preferred Reporting Items for Systematic review and Meta-Analysis Protocols) 2015 checklist: recommended items to address in a systematic review protocol (*with modifications in italics*)*

Section and topic	Item No	Checklist item	Section(s)
**ADMINISTRATIVE INFORMATION**			
Title:			
Identification	1a	Identify the report as a protocol of a systematic review *or the name of other review type*	Abstract
Update	1b	If the protocol is for an update of a previous systematic review, identify as such	n/a
Registration	2	If registered, provide the name of the registry (such as PROSPERO) and registration number	Abstract<br>Methods
Authors:			

Contact	3a	Provide name, institutional affiliation, e-mail address of all protocol authors; provide physical mailing address of corresponding author	Title
Contributions	3b	Describe contributions of protocol authors and identify the guarantor of the review	Title
Amendments	4	If the protocol represents an amendment of a previously completed or published protocol, identify as such and list changes; otherwise, state plan for documenting important protocol amendments	n/a
Support:			
Sources	5a	Indicate sources of financial or other support for the review	Abstract
Sponsor	5b	Provide name for the review funder and/or sponsor	Abstract
Role of sponsor or funder	5c	Describe roles of funder(s), sponsor(s), and/or institution(s), if any, in developing the protocol	2.5
**INTRODUCTION**			
Rationale	6	Describe the rationale for the review in the context of what is already known<br>*Provide a rationale for the choice of conducting the evidence synthesis method or approach. **	Introduction
Objectives	7	Provide an explicit statement of the question(s) the review will address with reference to participants, interventions, comparators, and outcomes (PICO)<br>*For Scoping Reviews JBI's population, concept and context (PCC) may be used*	2.2
**METHODS**			
Eligibility criteria	8	Specify the study characteristics (such as PICO, study design, setting, time frame) and report characteristics (such as years	2.3

		considered, language, publication status) to be used as criteria for eligibility for the review<br><br>*For Scoping Reviews JBI's population, concept and context (PCC) may be used **	
Information sources	9	Describe all intended information sources (such as electronic databases, contact with study authors, trial registers or other grey literature sources) with planned dates of coverage	2.3.3<br>2.4<br>2.4.1
Search strategy	10	Present draft of search strategy to be used for at least one electronic database, including planned limits, such that it could be repeated	Supp File 2.
Study records:			
Data management	11a	Describe the mechanism(s) that will be used to manage records and data throughout the review	2.4.1
Selection process	11b	State the process that will be used for selecting studies (such as two independent reviewers) through each phase of the review (that is, screening, eligibility and inclusion in meta-analysis *or other synthesis*)	2.4.1
Data collection process	11c	Describe planned method of extracting data from reports (such as piloting forms, done independently, in duplicate), any processes for obtaining and confirming data from investigators	2.4.1
Data items	12	List and define all variables for which data will be sought (such as PICO, *PCC or PPC* items, funding sources), any pre-planned data assumptions and simplifications	n/a
Outcomes and prioritization	13	List and define all outcomes *(if applicable)* for which data will be sought, including prioritization of main and additional outcomes, with rationale<br><br>*List and define other data items that will be sought **	n/a
Risk of bias in individual studies	14	Describe anticipated methods for assessing risk of bias of individual studies, including whether this will be done at the outcome or study level, or both; state how this information will be used in data synthesis	2.4.2

		*When applicable, state the rationale for not conducting a risk of bias assessment **	
Data synthesis	15a	Describe criteria under which study data will be quantitatively synthesised<br><br>*Describe criteria under which study data will be presented **	2.4.3
	15b	If data are appropriate for quantitative synthesis, describe planned summary measures, methods of handling data and methods of combining data from studies, including any planned exploration of consistency (such as I^2, Kendall's τ)<br><br>*Describe the planned approach for how extracted data will be presented (e.g., figures, tables, evidence gaps, maps) **	2.4.3
	15c	Describe any proposed additional analyses (such as sensitivity or subgroup analyses, meta-regression)<br><br>*Describe any proposed additional analyses (e.g., thematic analysis) **	2.4.3
	15d	If quantitative synthesis is not appropriate, describe the type of summary *or synthesis* planned	2.4.3
Meta-bias(es)	16	Specify any planned assessment of meta-bias(es) (such as publication bias across studies, selective reporting within studies)	n/a
Confidence in cumulative evidence	17	Describe how the strength of the body of evidence will be assessed (such as GRADE)	n/a

From: Shamseer L, Moher D, Clarke M, Ghersi D, Liberati A, Petticrew M, Shekelle P, Stewart L, PRISMA-P Group. Preferred reporting items for systematic review and meta-analysis protocols (PRISMA-P) 2015: elaboration and explanation. BMJ. 2015 Jan 2;349(jan02 1):g7647.

**Modifications align with:* Peters MDJ, Godfrey C, McInerney P, Khalil H, Larsen P, Marnie C, Pollock D, Tricco AC, Munn Z. Best practice guidance and reporting items for the development of scoping review protocols. JBI Evid Synth. 2022 Apr 1;20(4):953-968. doi: 10.11124/JBIES-21-00242. PMID: 35102103.
