# Supplementary material for: Protocol for a scoping review of traditional medicine research methods, methodologies, frameworks and strategies
Source: Front Med (Lausanne). 2024 Jul 10;11:1409392. doi: 10.3389/fmed.2024.1409392 (PMC11267516; doi:10.3389/fmed.2024.1409392)
Supplement: Supplementary file 2 [file Data_Sheet_2.pdf]

frontiers

Supplementary Material 2

Protocol for an Evidence Review of Traditional Medicine Research Methods, Methodologies, Frameworks and Strategies.

Nadine Ijaz*†, Jennifer Hunter†, Suzanne Grant, Kate Templeman

* Correspondence: Nadine.Ijaz@Carleton.ca

†These authors contributed equally to this work and share first authorship

2 Search terms and rationale

2.1 Rationale for the English-language TM search terms

The operational definition of complementary, alternative and integrative medicine, associated comprehensive search string of keywords and phrases presented by Ng et al. (1), and the traditional medicine (TM) typology proposed by Ijaz (2), were used as a starting point for selecting the TM search terms for this Review. Their rationales provided a valuable vantage point to iteratively develop consensus about which terms to include in the search strategy.

Due to pragmatic constraints, a balance between sensitivity and specificity was required to ensure epistemological congruency between the key TM systems, approaches, and modalities. In line with the Review, the focus was TM epistemologies rather than: a) any epistemology that does not align with conventional biomedical epistemology; or b) non-pharmacological supportive/adjuvant therapies that, like many TM approaches and modalities, are not well suited to being evaluated using the gold-standard, double-blind, randomized controlled trial study design. We deliberately discriminated between very broad terms for botanicals and nutraceuticals (e.g., herbal medicine, dietary supplements, probiotic) versus highly specific terms (e.g., turmeric, fish oil, niacin, lactobacilli) as preliminary database searches suggested that this would be sufficient to reach data saturation. Similarly, we excluded terms for non-specific therapies that may serve as both TM therapies in one context and biomedicine in another (3), and any term where preliminary database searches found a large proportion of the search results were not relevant to TM.

It is important to note that this inclusion/exclusion criteria only pertains to the terms that will be searched in English-language databases and not the Review's inclusion/exclusion criteria for evidence sources.

The inclusion criteria for the search terms were:

1. Overarching terms for Indigenous/TM systems (e.g., traditional Chinese medicine, homeopathy, Ayurvedic, Unani, yoga), including terms for Indigenous nations/tribes (e.g., Bwiti);
2. Terms for medical systems that draw strongly on TM epistemology (e.g., naturopathy, anthroposophy, chiropractic, osteopathy); and
3. Broad terms for modalities within these TM systems (e.g., cupping, acupuncture, herbal medicine, megavitamins, aromatherapy, apitherapy).

The exclusion criteria and brief rationale for the search terms were:

1. Terms for specific diets/dietary therapies (e.g., gluten-free diet, diet therapy, high fibre diet) that reflected a combination of pragmatic constraints and/or the term was not specific to TM;
2. Terms for individual ingredients/formulations (e.g., isoflavones, melatonin, cannabinoids) that reflected pragmatic constraints because there were too many individual terms and/or the term was not specific to TM;
3. Terms for systems or therapies that were 'invented' or were named (including registered/copyright names) during the 20th-21st Centuries (e.g., biodynamic therapy, Bowen technique, Feldenkrais Method), the focus is TM paradigms, rather than any paradigm that is not biomedical;
4. Terms for therapies, modalities, and supportive practices commonly delivered by allied health practitioners/services that do not directly draw on TM epistemology (e.g., rehabilitation, supportive care, social services/support, and various manual, physical, exercise/movement, diet, psychological, addiction, and occupational/expressive/pet therapies), as the focus is TM epistemology, not any non-pharmacological supportive/adjuvant therapy;
5. Non-specific terms that can apply to both TM and non-TM therapies, modalities, and practices (e.g., environmental medicine, hypnotherapy, laser therapy) that reflected pragmatic constraints; and

6. Martial arts that are predominantly a 'sport' (e.g., karate, kung fu) as the Review focus is therapies/medicine.

In the first instance, a list of 1,561 unique terms reflecting 604 therapeutic categories that were listed in Ng et al. (1) Table 2 were compiled into a Microsoft Excel spreadsheet. Single reviewers (JH and SG) independently screened the terms as per the above inclusion/exclusion criteria that were then checked by reviewer KT. Consensus decision making was used to resolve any disagreements and ensure credibility of the search terms. The final search terms represented the research team's expert views that were strongly held and/or commonly accepted.

Of the 604 therapeutic categories identified by Ng et al. (1), 127 were eventually included. From here, the unique terms in each category were pilot tested on the US National Library of Medicine's PubMed database and refined. This process highlighted preliminary frequent/infrequent and/or common therapy pairings.

The included Medicine Medical Subject Headings (MeSH) terms were selected by cross-checking the included key words and phrases against the National Library of Medicine MeSH descriptors to determine their place(s) in the MeSH tree structure, from which only the overarching MeSH categories were included. For example, the MeSH category "Acupuncture therapy" was not included because it is only situated under "Complementary Therapies", whilst "Medicine, traditional" was included because it is situated under both "Complementary Therapies" and "Culture".

To ensure optimize retrieval of sources that use both "Traditional" and "medicine" or "knowledge" the adjacent/near search operator will be used.

The following final listing of the TM search terms comprised of six main groupings that will be used during the database searches.

2.2 Search term examples

	**OVID PLATFORM**<br>AMED (Allied and Complementary Medicine); EBM Reviews - Cochrane Methodology Register; Ovid MEDLINE(R) ALL; APA PsycInfo
1	exp traditional medicine/ or exp Medicine, Traditional/ or exp Complementary Therapies/ or exp Integrative Medicine/

2	(Traditional adj5 medicin*).ab,ti.
3	("complementary medicin*" or "complementary therap*" or "traditional heal*" or "alternative medicin*" or "sectarian medicin*" or "ancient medicin*" or "folk medicin*" or "Ancestral Medicine*" or "Millenary" or "natural medicin*" or "Mindbody Medicin*" or "Mind body medicin*" or "integrative medicin*" or "integrative health*" or "integrative therap*" or "whole medical systems" or "clinical whole systems").ab,ti.
4	1 or 2 or 3
5	exp yoga/
6	("Anthroposophic Medicin*" or "Anthrophysical Medicin*" or "Arabic Medicin*" or "Ayurved*" or "ayush" or "Chiropract*" or "Chirotherapy" or "Homeopath*" or "Homoeopath*" or "Homotoxicology" or "Islamic Medicin*" or ("Mysticism" and "Heal*") or "Naturopathy" or "Naturopath" or "Naturopathic" or "Osteopath*" or "Persian Medicin*" or "Siddha" or "Tibetan Medicin*" or "Traditional Asian Medicin*" or "Traditional Korean Medicin*" or "Traditional Japanese Medicin*" or "Traditional Oriental Medicin*" or "Meridian System" or "Traditional Bhutanese Medicin*" or "Traditional Chinese Medicin*" or "Traditional East Asian Medicin*" or "Traditional European Medicin*" or "Traditional Indian Medicin*" or "Traditional Malay Medicin*" or "Traditional Mongolian" or "Mongolian Medicin*" or "Unani" or "Graeco-Arabic Medicin*" or "Yunani" or ("Witchcraft" and "therap*") or "Yoga" or "Yogic" or "Yogoda" or "Samyama").ab,ti.
7	5 or 6
8	(Bush adj3 medicin*).ab,ti.
9	(Huna adj5 Hawaii).ab,ti.
10	("Traditional healer*" or "Traditional healing" or "Traditional Maori" or "Aboriginal Medicin*" or "Bush Medicin*" or "Indigenous healing" or "Indigenous healer*" or "Indigenous medicin*" or "Kahuna" or "Native American Faith Healing" or "Native American Medicin*" or "Rongoa" or "Traditional birth attendant*" or "Traditional midwi*" or "Traditional South American" or "Traditional Tongan").ab,ti.
11	8 or 9 or 10
12	exp Ethnopharmacology/ or exp Herbal Medicine/
13	("Ethnomedicin*" or "Ethnopharm*" or "Ethnobiology" or "Botanicals" or "Dietary supplements" or "dietary supplement" or "Herbal Medicin*" or "Herbal Remed*" or "Herbal

	Tea" or "Tisane" or "Herbalism" or "Jamu" or "Megavitamin" or "Megavitamins" or "Nutraceutical" or "nutraceuticals" or "neutraceutical" or "neutraceuticals" or "Phytotherap*" or "Phytomedicin*" or "Phytoceutical*" or "Plant-Based Medicin*" or "Plant Based Medicin*" or "Prebiotics" or "prebiotic" or "Probiotics" or "probiotic").ab,ti.
14	12 or 13
15	exp traditional medicine practitioners/
16	(Sacred adj3 healing).ab,ti.
17	(esoteric adj3 therap*).ab,ti.
18	("Buddhist Tantric Practice" or "Bwiti" or "Calligraphy Therapy" or "Cymatic Therapy" or ("Cymatic" and "Therapy") or "Dervish danc*" or "Dukun" or "Ear Candling" or "ear candles" or "ear candle" or "Ear Coning" or "Essence therap*" or "Flor Essence" or "Flower Essence" or "floral therap*" or "Flower remed*" or "Gso-Ba Rig-Pa" or "Gsoba Rig-Pa" or "Gso-Ba Rigpa" or "Gurah" or "Initiatory Medicin*" or "Initiatory Treatment*" or "Initatory Therap*" or "Jala Neti" or "Neti Pot" or "Pot de Neti" or "Kneipp Cure" or "Mudras" or "Nuad Bo Rarn" or "Pranic healing" or "Primitive Medicin*" or "Psychic Medicin*" or "Psychic healing" or "Psychic healer" or "Sound healing" or ("Sufi" and ("whirl" or "whirled" or "whirling" or "whirls")) or "Sufi Healing" or "Thomsonianism" or "Traditional Cautery" or "Wild Medicine").ab,ti.
19	15 or 16 or 17 or 18
20	exp meditation/
21	Meditation/
22	("Abdominal Meridian Massage" or "Foot Reflexion Massage" or "Hot Stone Massage" or "Lomilomi Massage" or "Swedish Massage" or "Thai Massage" or "Tibetan Massage" or "Acupuncture" or "Acustimulation" or "acupressure" or "acupoint stimulation" or "Acumoxa" or "Acupression" or "Electroacupuncture" or "Catgut Implantation" or "moxibustion" or "Amma" or "Ammotherapy" or "Ammo Therapy" or "Psammotherapy" or "sand therapy" or "sand-therapy" or "Animistic" or "Animism" or "Anma" or "Traditional Japanese Massage" or "Apitherapy" or "Apipuncture" or "Apis Mellifera Venom" or "Apiterapia" or "Api-Treatment" or "Apitherapie" or "Bee Sting Therapy" or "Bee Therapy" or "Bee Venom Therapy" or "Honeybee product" or "Aromatherapy" or "Aroma Therapy" or "Aroma Treatment" or "Aromaterapia" or "Aromatherapie" or "Aromatic Therapy" or "Auriculotherapy" or "Auricular Therapy" or "Balneotherapy" or "Balneological Treatment"

	or "Balneotherapeutics" or "Crenobalneotherapy" or "Therapeutic Bathing" or "Medical Hydrology" or "Social Thermalism" or "Bone setting" or "Traditional Bone Setting" or "Chanting" or "Crystal Therapy" or "Crystal Healing" or "Gem Therapy" or "Gemstone Therapy" or "Cupping" or "Hijamat" or "Pneumatic Pulsation Therapy" or "Curanderismo" or "Curandera" or "Curandero" or "Folk Healers" or "Latin American Folk Medicine" or "feng shui" or "Fengshui" or "Fusui" or "Guided Imagery" or "Guided Visualization" or "Healing touch" or "Heliotherap*" or "Hirudotherapy" or "Hirudin" or "Leech Therapy" or "Medicinal Leeches" or "Home Remedies" or "Iridology" or "Kampo" or "Maggot Therapy" or "Larva Therapy" or "Larval Debridement Therapy" or "Marma" or "Meditation" or "Samadhi" or "Acu Moxi" or "Dogbi" or "Moxa" or "Okyu" or "Mud Therapy" or "Peloid" or "Panchakarma" or "Prophetic Medicin*" or "Qi Gong" or "Ba Duan Jin" or "Biyun" or "Baduanjin" or "Chi Gung" or "Chi Kung" or "Dantian" or "Guolin" or "Hua Gong" or "Qigong" or "Wuqinxi" or "Reflexology" or "Sauna" or "steam bath" or "Scraping" or "Gua Sha" or "Shamanism" or "Samanism" or "Shiatsu" or "Spa therap*" or "Speleotherap*" or "Tai chi" or "Taichi" or "Taijiquan" or "Taiji" or "Tai Ji" or "thalassotherapy" or "Therapeutic Touch" or "Touch Therap*" or "Tui Na" or "Tuina" or "Urine therap*" or "Amaroli" or "AutoUrine Therap*" or "AutoUrotherap*" or "Mutra Paribhasa" or "Mutra Varga" or "Naramutra" or "Shivambu" or "Urotherap*" or "Voodoo").ab,ti.
23	20 or 21 or 22
24	4 or 7 or 11 or 14 or 19 or 23
25	research methodolog*.ab,ti.
26	methodological approach*.ab,ti.
27	methodolog *.ti.
28	("realist" or "program theory" or "program logic" or "logic analysis" or "model theory" or "logic model*" or "mixed method" or "systems theor*" or "Complex dynamic system" or "complexity theor*" or "Complex adaptive system" or "complexity science" or "system* research" or "implementation research" or "implementation science").ab,ti.
29	*Research Report/st [Standards]
30	guideline/
31	Effectiveness guidance document.ab,ti.
32	Risk of Bias.ti.

33	(("research" or "Method*" or "model") and ("guidance" or "guideline*")).ti.
34	("Framework*" or "Consensus").ti.
35	(research adj5 framework*).ab,ti.
36	(("Reporting" or "research") and ("checklist*" or "guideline")).ti.
37	("research" and "standard*").ti.
38	("core outcome set" or "research strateg*").ab,ti.
39	epistemic.ab,ti.
40	epistemolog*.ab,ti.
41	("ways of knowing" or "two-eyed seeing").ab,ti.
42	(research adj5 paradigm*).ab,ti.
43	(therap* adj5 paradigm*).ab,ti.
44	("fit-for-purpose" or "fit for purpose").ab,ti.
45	Model valid*.ab,ti.
46	25 or 26 or 27 or 28 or 29 or 30 or 31 or 32 or 33 or 34 or 35 or 36 or 37 or 38 or 39 or 40 or 41 or 42 or 43 or 44 or 45
47	24 and 46
48	("whole system research" or "whole systems research").ab,ti.
49	("Indigenous method*" or "Indigenous research methodolog*" or "Indigenist research methodolog*").ab,ti.
50	48 or 49
51	47 or 50

**EBSCOHOST PLATFORM**<br>CINAHL; Anthropology Plus; Humanities Source Ultimate; Psychology and Behavioral Sciences Collection; Sociology Source Ultimate.	
S1	TI ("complementary medicin*" or "complementary therap*" or "traditional heal*" or "alternative medicin*" or "sectarian medicin*" or "ancient medicin*" or "folk medicin*" or "ancestral medicin*" or “millenary” or "natural medicin*" or "mind body medicin*" or "mindbody medicine" or "integrative medicin*" or "integrative health*" or "integrative therap*" or "whole medical systems" or "clinical whole systems") OR AB ("complementary medicin*" or "complementary therap*" or "traditional heal*" or

	"alternative medicin*" or "sectarian medicin*" or "ancient medicin*" or "folk medicin*" or "ancestral medicin*" or “millenary” or "natural medicin*" or "mind body medicin*" or "mindbody medicin*" or "integrative medicin*" or "integrative health*" or "integrative therap*" or "whole medical systems" or "clinical whole systems")
S2	TI Traditional N5 (medicine or medicines) OR AB Traditional N5 (medicine or medicines)
S3	(MH "Alternative Therapies+") or (MH "Medicine, Traditional+") or (MH "Integrative Medicine+")
S4	(DE "TRADITIONAL medicine") or (DE "ALTERNATIVE medicine") or (DE "INTEGRATIVE medicine")
S5	S1 OR S2 OR S3 OR S4
S6	TI ("anthroposophic medicin*" or "anthrophysical medicin*" or "arabic medicin* or "ayurved*" or "ayush" or "chiropract*" or "chirotherapy" or "homeopath*" or "homoeopath*" or "homotoxicology" or "islamic medicin*" or ("mysticism" and "heal*") or "naturopathy" or "naturopath" or "naturopathic" or "osteopath*" or “persian medicin*" or "siddha" or “tibetan medicin*” or "traditional asian medicine" or "traditional korean medicin*" or "traditional japanese medicin*" or "traditional oriental medicin*" or "meridian system" or "traditional bhutanese medicin*" or "traditional chinese medicin*" or "traditional east asian medicin*" or "traditional european medicin*" or "traditional indian medicin*" or "traditional malay medicin*" or "traditional mongolian" or "mongolian medicin*" or "unani" or "graeco-arabic medicin*" or "yunani" or (“witchcraft" and "therap*") or "yoga" or "yogic" or "yogoda" or "samyama") OR AB "anthroposophic medicin*" or "anthrophysical medicin*" or "arabic medicin* or "ayurved*" or "ayush" or "chiropract*" or "chirotherapy" or "homeopath*" or "homoeopath*" or "homotoxicology" or "islamic medicin*" or ("mysticism" and "heal*") or "naturopathy" or "naturopath" or "naturopathic" or "osteopath*" or “persian medicin*" or "siddha" or “tibetan medicin*” or "traditional asian medicine" or "traditional korean medicin*" or "traditional japanese medicin*" or "traditional oriental medicin*" or "meridian system" or "traditional bhutanese medicin*" or "traditional chinese medicin*" or "traditional east asian medicin*" or "traditional european medicin*" or "traditional indian medicin*" or "traditional malay medicin*" or "traditional mongolian" or

	"mongolian medicin*" or "unani" or "graeco-arabic medicin*" or "yunani" or (“witchcraft" and "therap*") or "yoga" or "yogic" or "yogoda" or "samyama")
S7	(MH "Yoga+")
S8	S6 OR S7
S9	TI (“traditional healer” or “traditional healing” or "traditional maori" or "aboriginal medicin*" or "bush medicin*" or “indigenous healing” or "Indigenous healer*" or "indigenous medicin*" or "kahuna" or "native american faith healing" or "native american medicin*" or "rongoa" or "traditional birth attendant*" or "traditional midwi*" or "traditional south american" or "traditional tongan") OR AB (“traditional healer” or “traditional healing” or "traditional maori" or "aboriginal medicin*" or "bush medicin*" or “indigenous healing” or "Indigenous healer*" or "indigenous medicin*" or "kahuna" or "native american faith healing" or "native american medicin*" or "rongoa" or "traditional birth attendant*" or "traditional midwi*" or "traditional south american" or "traditional tongan")
S10	TI bush N3 (medicine or medicines) OR AB bush (medicine or medicines) OR TI Huna N3 Hawaii OR AB Huna N3 Hawaii
S11	S9 OR S10
S12	TI ("ethnomedicin*" or “ethnopharm*” or "ethnobiology" or "botanicals" or "dietary supplements" or "dietary supplement" or "herbal medicin*" or "herbal remed*" or "herbal tea" or "tisane" or "herbalism" or "jamu" or "megavitamin" or "megavitamins" or "nutraceutical" or "nutraceuticals" or "neutraceutical" or "neutraceuticals" or "phytotherap*" or "phytomedicin*" or "phytoceutical*" or "plant-based medicin*" or "plant based medicin*" or "prebiotics" or "prebiotic" or "probiotics" or "probiotic") OR AB ("ethnomedicin*" or “ethnopharm*” or "ethnobiology" or "botanicals" or "dietary supplements" or "dietary supplement" or "herbal medicin*" or "herbal remed*" or "herbal tea" or "tisane" or "herbalism" or "jamu" or "megavitamin" or "megavitamins" or "nutraceutical" or "nutraceuticals" or "neutraceutical" or "neutraceuticals" or "phytotherap*" or "phytomedicin*" or "phytoceutical*" or "plant-based medicin*" or "plant based medicin*" or "prebiotics" or "prebiotic" or "probiotics" or "probiotic")
S13	(MH "Medicine, Herbal+")
S14	(DE "ETHNOPHARMACOLOGY")
S15	S12 OR S13 OR S14

S16	TI ("abdominal meridian massage" or "foot reflexion massage" or "hot stone massage" or "lomilomi massage" or "swedish massage" or "thai massage" or "tibetan massage" or "acupuncture" or "acustimulation" or "acupressure" or "acupoint stimulation" or "acumoxa" or "acupression" or "electroacupuncture" or "catgut Implantation" or "moxibustion" or "amma" or "ammotherapy" or "ammo therapy" or "psammotherapy" or "sand therapy" or "sand-therapy" or "animistic" or "animism" or "anma" or "traditional japanese massage" or "apitherapy" or "apipuncture" or "apis mellifera venom" or "apiterapia" or "api-treatment" or "apitherapie" or "bee sting therapy" or "bee therapy" or "bee venom therapy" or "honeybee product" or "aromatherapy" or "aroma therapy" or "aroma treatment" or "aromaterapia" or "aromatherapie" or "aromatic therapy" or "auriculotherapy" or "auricular therapy" or "balneotherapy" or "balneological treatment" or "balneotherapeutics" or "crenobalneotherapy" or "therapeutic bathing" or "medical hydrology" or "social thermalism" or "bone setting" or "traditional bone setting" or "chanting" or "crystal therapy" or "crystal healing" or "gem therapy" or "gemstone therapy" or "cupping" or "hijamat" or "pneumatic pulsation therapy" or "curanderismo" or "curandera" or "curandero" or "folk healers" or "latin american folk medicine" or "feng shui" or "fengshui" or "fusui" or “guided imagery" or "guided visualization" or "healing touch" or "heliotherap*” or "hirudotherapy" or "hirudin" or "leech therapy" or "medicinal leeches" or "home remedies" or "iridology" or "kampo" or "maggot therapy" or "larva therapy" or "larval debridement therapy" or "marma" or "meditation" or "samadhi" or "acu moxi" or "dogbi" or "moxa" or "okyu" or "mud therapy" or "peloid" or "panchakarma" or "prophetic medicin*" or "qi gong" or "ba duan jin" or "biyun" or "baduanjin" or "chi gung" or "chi kung" or "dantian" or "guolin" or "hua gong" or "qigong" or "wuqinxi" or "reflexology" or "sauna" or "steam bath" or "scraping" or "gua sha" or "shamanism" or "samanism" or "shiatsu" or "spa therap*" or "speleotherap*" or "tai chi" or "taichi" or "taijiquan" or "taiji" or "tai ji" or "thalassotherapy" or "therapeutic touch" or "touch therap*" or "tui na" or "tuina" or "urine therap*" or "amaroli" or "autourine therap*" or "autourotherap*" or "mutra paribhasa" or "mutra varga" or "naramutra" or "shivambu" or "urotherap*" or "urotherap*" or "voodoo") OR AB ("abdominal meridian massage" or "foot reflexion massage" or "hot stone massage" or "lomilomi massage" or "swedish massage" or "thai massage" or "tibetan massage" or

	"acupuncture" or "acustimulation" or "acupressure" or "acupoint stimulation" or "acumoxa" or "acupression" or "electroacupuncture" or "catgut Implantation" or "moxibustion" or "amma" or "ammotherapy" or "ammo therapy" or "psammotherapy" or "sand therapy" or "sand-therapy" or "animistic" or "animism" or "anma" or "traditional japanese massage" or "apitherapy" or "apipuncture" or "apis mellifera venom" or "apiterapia" or "api-treatment" or "apitherapie" or "bee sting therapy" or "bee therapy" or "bee venom therapy" or "honeybee product" or "aromatherapy" or "aroma therapy" or "aroma treatment" or "aromaterapia" or "aromatherapie" or "aromatic therapy" or "auriculotherapy" or "auricular therapy" or "balneotherapy" or "balneological treatment" or "balneotherapeutics" or "crenobalneotherapy" or "therapeutic bathing" or "medical hydrology" or "social thermalism" or "bone setting" or "traditional bone setting" or "chanting" or "crystal therapy" or "crystal healing" or "gem therapy" or "gemstone therapy" or "cupping" or "hijamat" or "pneumatic pulsation therapy" or "curanderismo" or "curandera" or "curandero" or "folk healers" or "latin american folk medicine" or "feng shui" or "fengshui" or "fusui" or “guided imagery" or "guided visualization" or "healing touch" or "heliotherap*” or "hirudotherapy" or "hirudin" or "leech therapy" or "medicinal leeches" or "home remedies" or "iridology" or "kampo" or "maggot therapy" or "larva therapy" or "larval debridement therapy" or "marma" or "meditation" or "samadhi" or "acu moxi" or "dogbi" or "moxa" or "okyu" or "mud therapy" or "peloid" or "panchakarma" or "prophetic medicin*" or "qi gong" or "ba duan jin" or "biyun" or "baduanjin" or "chi gung" or "chi kung" or "dantian" or "guolin" or "hua gong" or "qigong" or "wuqinxi" or "reflexology" or "sauna" or "steam bath" or "scraping" or "gua sha" or "shamanism" or "samanism" or "shiatsu" or "spa therap*" or "speleotherap*" or "tai chi" or "taichi" or "taijiquan" or "taiji" or "tai ji" or "thalassotherapy" or "therapeutic touch" or "touch therap*" or "tui na" or "tuina" or "urine therap*" or "amaroli" or "autourine therap*" or "autourotherap*" or "mutra paribhasa" or "mutra varga" or "naramutra" or "shivambu" or "urotherap*" or "urotherap*" or "voodoo")
S17	(MH "Relaxation Techniques+")
S18	(DE "MEDITATION" or DE "TRANSCENDENTAL Meditation")
S19	S16 SO S17 OR S18
S20	TI ("buddhist tantric practice" or "bwiti" or "calligraphy therapy" or "cymatic therapy" or ("cymatic" and "therapy") or "dervish danc*" or "dukun" or "ear candling" or "ear

	candles" or "ear candle" or "ear coning" or "essence therap*" or "flor essence" or "flower essence" or "floral therap*" or "flower remed*" or "gso-ba rig-pa" or "gsoba rig-pa" or "gso-ba rigpa" or "gurah" or "initiatory medicin*" or "initiatory treatment*" or "initiatory therap*" or "jala neti" or "neti pot" or "pot de neti" or "kneipp cure" or "mudras" or "nuad bo rarn" or "pranic healing" or "primitive medicin*" or "psychic medicin*" or "psychic healing" or "psychic healer" or "sound healing" or ("sufi" and ("whirl" or "whirled" or "whirling" or "whirls")) or "Sufi healing" or "thomsonianism" or "traditional cautery" or "wild medicine") OR AB ("buddhist tantric practice" or "bwiti" or "calligraphy therapy" or "cymatic therapy" or ("cymatic" and "therapy") or "dervish danc*" or "dukun" or "ear candling" or "ear candles" or "ear candle" or "ear coning" or "essence therap*" or "flor essence" or "flower essence" or "floral therap*" or "flower remed*" or "gso-ba rig-pa" or "gsoba rig-pa" or "gso-ba rigpa" or "gurah" or "initiatory medicin*" or "initiatory treatment*" or "initiatory therap*" or "jala neti" or "neti pot" or "pot de neti" or "kneipp cure" or "mudras" or "nuad bo rarn" or "pranic healing" or "primitive medicin*" or "psychic medicin*" or "psychic healing" or "psychic healer" or "sound healing" or ("sufi" and ("whirl" or "whirled" or "whirling" or "whirls")) or "Sufi healing" or "thomsonianism" or "traditional cautery" or "wild medicine")
S21	(IT esoteric N3 therap*) or (AB esoteric N3 therap*) or (TI sacred N3 healing) or (AB sacred N3 healing)
S22	(MH "Traditional Healers") or (MH "Alternative Health Personnel+")
S23	S20 OR 21 OR S22
S24	S5 OR S8 OR S11 OR S15 OR S19 OR 23
S25	TI (“research methodolog*”) or AB (“research methodolog*”)
S26	TI (“methodological approach*”) or AB (“methodological approach*”)
S27	TI (methodolog*)
S28	TI ("realist" or "program theory" or "program logic" or "logic analysis" or "model theory" or "logic model*" or "mixed method" or "systems theor*" or "complex dynamic system" or "complexity theor*" or "complex adaptive system" or "complexity science" or "system* research" or "implementation research" or "implementation science") OR AB ("realist" or "program theory" or "program logic" or "logic analysis" or "model theory" or "logic model*" or "mixed method" or "systems theor*" or "complex dynamic system" or

	"complexity theor*" or "complex adaptive system" or "complexity science" or "system* research" or "implementation research" or "implementation science")
S29	(MH "Research Reports+/ST")
S30	(MH "Guideline+")
S31	TI (“effectiveness guidance document”) or AB (“effectiveness guidance document”)
S32	TI (“risk of bias”)
S33	TI (("research" or "method*" or "model") and ("guidance" or "guideline*"))
S34	TI (“framework*”) or TI (“consensus“)
S35	TI research N5 framework* or AB research N5 framework*
S36	TI (("reporting" or "research") and ("checklist*" or "guideline*”))
S37	TI ("research" and "standard*")
S38	TI ("core outcome set" or "research strateg*") or AB ("core outcome set" or "research strateg*")
S39	TI (“epistemic”) or AB (“epistemic”)
S40	TI (“epistemolog*”) or AB (“epistemolog*”)
S41	TI (“ways of knowing” or “two-eyed seeing”) or AB (“ways of knowing” or two-eyed seeing”)
S42	TI (research N5 paradigm* or therap* paradigm*) or AB (research N5 paradigm* or therap* paradigm*)
S43	TI (“fit-for-purpose” or “fit for purpose”) or AB (“fit-for-purpose’’ or “fit for purpose”)
S44	TI (“model valid*”) or AB (“model valid*”)
S45	S25 OR S26 OR S27 OR S28 OR S29 OR S30 OR S31 OR S32 OR S33 OR S34 OR S35 OR S36 OR S37 OR 38 OR 39 OR 40 OR 41 OR 42 OR 43 OR 44
S46	S24 AND S45
S47	TI (“whole system research” or “whole systems research”) or AB (“whole system research” or “whole systems research”)
S48	TI ("Indigenous method*" or "Indigenous research methodolog*" or "Indigenist research methodolog*") OR AB ("Indigenous method*" or "Indigenous research methodolog*" or "Indigenist research methodolog*")
S49	S47 OR S48
S50	S46 OR S49

GLOBAL INDEX MEDICUS PLATFORM<br>LILACS (Americas); WPRIM (Western Pacific); IMEMR (Eastern Mediterranean); IMSEAR (South-East Asia); AIM (Africa)
tw:((af:(tw:((af:((mh:mt*) OR (mh:hp*) OR (mh:e02.190*) OR (mh:i01.076.201.450.654*) OR (mh:h01.158.703.015) OR (mh:h02.628.100) OR (mh:h02.628.190))) OR (ti:(“anthroposophic” OR “anthroposophical” OR "Ayurveda" OR "Ayurvedic" OR "ayush" OR "Naturopathy" OR "Naturopaths" OR "Siddha" OR “unani” OR "Yoga" OR "Ethnomedicine" OR "Ethnopharmacology" OR "Ethnobiology" OR "Herbal" OR "Phytotherapy" OR "Phytotherapies" OR "Acupuncture" OR "Acustimulation" OR "acupressure" OR "Acumoxa" OR "Acupression" OR "Electroacupuncture" OR "moxibustion" OR "Aromatherapy" OR "Balneotherapy" OR "Kampo" OR "Meditation" OR "Qigong" OR "Reflexology" OR "Taichi" OR "Taijiquan" OR "Voodoo")) OR (ab:(“anthroposophic” OR “anthroposophical” OR "Ayurveda" OR "Ayurvedic" OR "ayush" OR "Naturopathy" OR "Naturopaths" OR "Siddha" OR “unani” OR "Yoga" OR "Ethnomedicine" OR "Ethnopharmacology" OR "Ethnobiology" OR "Herbal" OR "Phytotherapy" OR "Phytotherapies" OR "Acupuncture" OR "Acustimulation" OR "acupressure" OR "Acumoxa" OR "Acupression" OR "Electroacupuncture" OR "moxibustion" OR "Aromatherapy" OR "Balneotherapy" OR "Kampo" OR "Meditation" OR "Qigong" OR "Reflexology" OR "Taichi" OR "Taijiquan" OR "Voodoo"))))) AND (af:((ti:(“methodology” OR “methodological” OR “strategy” OR “framework” OR "realist" OR “consensus”)) OR (ti:(("Reporting" OR "research") AND ("checklist" OR "guideline"))))))

References

1. Ng JY, Dhawan T, Dogadova E, Taghi-Zada Z, Vacca A, Fajardo R-G, Masood HA, Patel R, Sunderji S, Wieland LS, et al. A comprehensive search string informed by an operational definition of complementary, alternative, and integrative medicine for systematic bibliographic database search strategies. *BMC Complement Med Ther* (2022) 22:200. doi: 10.1186/s12906-022-03683-1

2. Ijaz N. What is Traditional Medicine? A Typology for Operationalizing the World Health Organization Definition. (2023) http://dx.doi.org/10.2139/ssrn.4564463

3. Hunter J, Harnett JE, Chan W-JJ, Pirotta M. What is integrative medicine? Establishing the decision criteria for an operational definition of integrative medicine for general practice health services research in Australia. *Integr Med Res* (2023) 12:100995. doi: 10.1016/j.imr.2023.100995
