# Supplementary material for: Protocol for a scoping review of traditional medicine research methods, methodologies, frameworks and strategies
Source: Front Med (Lausanne). 2024 Jul 10;11:1409392. doi: 10.3389/fmed.2024.1409392 (PMC11267516; doi:10.3389/fmed.2024.1409392)
Supplement: Supplementary file 3 [file Data_Sheet_3.pdf]

Supplementary Material 3

Protocol for an Evidence Review of Traditional Medicine Research Methods, Methodologies, Frameworks and Strategies.

Nadine Ijaz*†, Jennifer Hunter†, Suzanne Grant, Kate Templeman

* Correspondence: Nadine.Ijaz@Carleton.ca

†These authors contributed equally to this work and share first authorship

3 Data extraction form

1. General information

1. 1.1 Document ID

1st author last name and year (e.g. Ijaz 2021) or organization abbreviation and year (e.g. WHO 2018)

[ ]

1.2 Year published

[ ]

1.3a Citation details

[ ]

1.3b Citation details (Merged article 2)

[ ]

1.3c Citation details (Merged article 3)

[ ]

1.3c Citation details (Merged article(s) 4+)

[ ]

1.4 DOI / URL

ideally the http:// hyperlink

1.5 Are the citation details in Covidence correct?

☐ Yes

- ☐ No (correct in EndNote)

1.6 Publication Language(s)

- ☐ English
- ☐ Chinese
- ☐ French
- ☐ German
- ☐ Japanese
- ☐ Korean
- ☐ Portuguese
- ☐ Spanish
- ☐ Thai
- ☐ Other [ ]

1.7 Country/Region of ALL authors

Select all that apply

- ☐ Australia
- ☐ Brazil
- ☐ Canada
- ☐ China
- ☐ Germany
- ☐ India
- ☐ Iran
- ☐ Japan
- ☐ South Korea
- ☐ Thailand
- ☐ UK
- ☐ USA
- ☐ Other [ ]

1.8 What type of document is this?

Documents not published in a peer review journal are Grey Literature.

If there are merged documents, select all that apply

- ☐ Primary study / research or protocol (published in a peer review journal)
- ☐ Secondary study / literature review or protocol (published in a peer review journal)
- ☐ Opinion, commentary (review), editorial (published in a peer review journal)
- ☐ Letter to editor / response letter (published in a peer review journal)
- ☐ Guideline (published in a journal OR Grey literature e.g., Government)
- ☐ Other article published in a peer review journal (specify in 'Other')

- ☐ Book or Book Chapter
- ☐ Research thesis / dissertation
- ☐ Other Grey literature e.g., report / white paper etc. (specify in ‘Other’)
- ☐ Website
- ☐ Other [ ]

1.9 If unsure about the document type - explain here:

[ ]

2. Declarations

2.1 Was funding and interests declared?

Note: declaring "no funding" = funding declared.

declaring "no conflicts of interest" = interests declared.

- ☐ Funding & interests declared
- ☐ Funding declared; interests not declared
- ☐ Interests declared; funding not declared
- ☐ Not declared
- ☐ N/A (published by Gov /org.)

2.2 (OPTIONAL) Details if funding declared

Either write: Declared no funding or provide details.

[ ]

2.3 (OPTIONAL) Summary if funding declared

Including who funded the research grant(s)

- ☐ No funding
- ☐ Government
- ☐ University
- ☐ Non-profit organization
- ☐ Industry / for-profit organization
- ☐ Other [ ]

2.4 (OPTIONAL) Details if interests declared

Either write: Declared no interests or provide details.

[ ]

3. Research domain / discipline

3.1 What research domain(s) or discipline(s) was applied, discussed or recommended?

Select all that apply

- ☐ Basic sciences / preclinical
- ☐ Ethnomedicine (e.g., ethnobotany / ethnopharmacology / medical anthropology)
- ☐ Indigenous research methodologies
- ☐ Clinical research
- ☐ Public health / health promotion / Epidemiology (health-related states in populations)
- ☐ Health services research / health economics
- ☐ Social sciences (including TM use, attitudes etc.) / policy or regulatory research
- ☐ Implementation science / translational science (including industry R&D)
- ☐ Informatics (e.g., databases, data mining, AI, TM characterization / characterization)
- ☐ Other

3.2 (OPTIONAL) Comments about the research domains

If you were unsure about your previous answer, please explain here to help us with the final coding.

4. TM Types

4.1 Which of the following traditional, complementary or integrative medicine (TM) was studied or discussed?

When relevant please also specify in 'OTHER'

Select all that apply.

- ☐ TM (i.e., general / not specific)
- ☐ Any TM whole system (i.e., not specific)
- ☐ Any TM product / herb (i.e., not specific)
- ☐ Any TM mind-body therapy / practice (i.e., not specific)
- ☐ Non-codified Indigenous healing practices
- ☐ Anthroposophical medicine
- ☐ Ayurveda
- ☐ Traditional East Asian Medicine
- ☐ Naturopathy
- ☐ Persian medicine
- ☐ Siddha
- ☐ Unani
- ☐ Chiropractic
- ☐ Osteopathy
- ☐ Manual / touch therapies

- ☐ Acupuncture other acupoint therapy
- ☐ Cupping
- ☐ Tai Chi / Qigong
- ☐ Yoga
- ☐ Meditation / mindfulness / contemplative practices
- ☐ Expressive therapies (e.g., art, music, drama)
- ☐ Spiritual / energetic healing (e.g., Reiki, Ngangkari, prayer, ceremony, rituals)
- ☐ Other 'mind-body' therapies
- ☐ Botanicals / herbal medicine
- ☐ Nutraceuticals (not herbs)
- ☐ Homeopathy
- ☐ Food as medicine (including social practices)
- ☐ Birthing practices
- ☐ Country/ land/ nature as healer/ therapy
- ☐ Other

4.2 (OPTIONAL) Comments about the TM types.

If you were unsure about your previous answer, please explain here to help us with the final coding.

5 Summary of research approaches

5.1 The document (or other evidence source) was included in this review because it is a:

Select all that apply

- ☐ DESCRIPTION / EXAMPLE: Summarizes or maps research methods, methodologies, guidelines etc. that have been used in TM (e.g., literature review, novel methods, or just an example).

- ☐ RATIONALE / FRAMEWORK: Explains why a research approach is suitable or unsuitable for studying TM, or suggests how a research approach could be modified to make it suitable for TM research.

- ☐ GUIDANCE: Provides formal or informal guidance or recommendations for how to conduct or report TM research (e.g., CONSORT or PRISMA TM extensions, National guidelines, other consensus statements).

- ☐ EXAMPLE OF PARADIGMATIC ALIGNMENT: A primary study, secondary study, guidance document etc. where the research approach applied or discussed was fit-for-purpose, appropriate or sensitive to the TM being studied (e.g., used TM diagnosis, individualized TM treatment, outcomes specific to TM, Indigenous or TM research methodology, mixed methods, real world evidence, herbal synergies, applies complexity science etc.) [NOTE: must answer next question]

5.2 (OPTIONAL) Comments reason for inclusion.

If you were unsure about your previous answer, please explain here to help us with the final coding.

5.3 This is relevant to the conceptual framework of paradigmatic alignment because the authors summarize, apply, discuss or recommend one or more of the following:

ONLY select if definitely relevant, otherwise select n/a
[NOTE: If this is an example, then tick the last option in the previous question]

- ☐ n/a
- ☐ Cultural responsiveness (e.g., cultural safety, co-design, local adaptation)
- ☐ Multi-component / multi-ingredient / multi-modal intervention
- ☐ Individualized / semi-standardized intervention (manualization)
- ☐ Comparison intervention / placebo / sham
- ☐ TM diagnosis / indication
- ☐ TM specific outcome measure
- ☐ TM specific guidance for conducting or synthesizing research
- ☐ TM specific guidance for reporting research
- ☐ TM specific quality appraisal (e.g., model validity/fidelity, RoB)
- ☐ Indigenous or TM research methodology
- ☐ Etuaptmumk (Two-Eyed Seeing), Ganma (Both-ways), Pluralism
- ☐ Model (fit) validity / fidelity
- ☐ Whole systems research
- ☐ Mixed methods
- ☐ Pragmatic or naturalistic trial / comparative effectiveness (aligns with TM practice)
- ☐ Real world evidence (including surveillance AEs etc.)
- ☐ Complexity science (e.g., AI, data mining, network pharmacology)
- ☐ Herbal synergies
- ☐ Novel preclinical methods
- ☐ Other

5.4 (OPTIONAL) Further details about how or why is this an example or discussion of paradigmatic alignment.

If you were unsure about your previous answer, please explain here to help us with the final coding.

5.5 The document (or other evidence source) was included in this review because it used, discussed or recommended the following research approach(es):

RESEARCH APPROACHES = METHOD: technique to gather, analyze or report data. METHODOLOGY: principles /procedures / study designs. FRAMEWORK: conceptual/theoretical rationale or approach to research. GUIDELINES: for ethical and rigorous conduct/reporting of research. STRATEGY: plans to conduct or support research, including R&D

- ☐ Research method / technique
- ☐ Research methodology / study design
- ☐ Research framework / rationale
- ☐ Guideline for conducting or reporting research
- ☐ Strategy for research
- ☐ Other ______

5.6 (OPTIONAL) Comments on the relevance of the document.

If you were unsure about how to answer, please explain here to help us with final the coding.

______

5.7 What was the aim or purpose of this research approach(es)?

Select all that apply

- ☐ IMPROVE or adapt the methods, methodology, conduct, quality or reporting of TM research.
- ☐ ENHANCE paradigm alignment between the research approach and TM being studied.
- ☐ DEVELOP the TM system, therapy or practice; or investigate how or why it works (e.g., preclinical, basic experimental studies (BESH) therapeutic framework, diagnostic methods/ reliability, treating or assessing outcomes etc.)
- ☐ DEMONSTRATE or prove that the TM is a 'science' (e.g., according to philosophy of science)
- ☐ SUMMARIZE or describe research approaches used to study a TM
- ☐ DOCUMENT Indigenous/ TM knowledge or research methodologies
- ☐ DESCRIBE or generate information about the people who use or provide a TM (e.g., ethnomedicine, surveys, interviews, social media research etc.)
- ☐ EVALUATE a TM system, intervention, service etc. (e.g., safety, effectiveness, acceptability, costs, funding, feasibility, implementation, post-market surveillance etc.)
- ☐ DISCOVER new applications and uses for TM (e.g., herbal drug discovery)
- ☐ INFORM decision-making (e.g., clinical care, health service provision, funding/insurance, regulation etc.)
- ☐ Other ______

5.8 (OPTIONAL) Comments on the purpose or aim of the document.

If you were unsure about how to answer, please explain here to help us with final coding.

5.9 (OPTIONAL) Document Purpose / Aim

"Quote" relevant excerpts or write in your own words

5.10 Shortlist for in-depth analysis or as a potential exemplar?

e.g. an exemplary method, methodology, framework, guideline, or strategy

- ☐ Definitely yes
- ☐ Probably not
- ☐ Definitely not
- ☐ Unsure

5.11 (OPTIONAL) Reason for shortlisting, not shortlisting or unsure

5.12 (OPTIONAL) Further details / comments

PLEASE GO TO THE RELEVANT SECTION

SECTION 6: PRIMARY STUDIES

SECTION 7: LITERATURE REVIEWS

SECTION 8: OTHER DOCUMENTS AND EVIDENCE SOURCES

NOTE: for a definition of BESH vs. Clinical Trial see:
https://www.ninds.nih.gov/sites/default/files/migrate-documents/guidance-for-besh-foas_508c.pdf

6 PRIMARY STUDY (OR PROTOCOL)

6.1 What was the methodology / study design?

Select any that apply

- ☐ UNSURE how to classify (please write details in Comments section)
- ☐ Basic sciences / Preclinical (non-specific, or write in 'Other')
- ☐ In silico / computer simulations / AI

- ☐ In vivo / in vitro
- ☐ Network pharmacology
- ☐ System / Complexity Sciences
- ☐ Basic Experimental Studies Involving Humans (BESH)
- ☐ Single arm feasibility or pilot study
- ☐ Randomized controlled trial (RCT)
- ☐ Non-randomized studies of interventions (NRSI)
- ☐ Case series / report
- ☐ N-of-1
- ☐ Other Clinical research (non-specific, or write in ‘Other’)
- ☐ Cross sectional study
- ☐ Other quantitative study (non-specific, or write in ‘Other’)
- ☐ Anthropology / ethnomedicine
- ☐ Ethnopharmacology / ethnobotany
- ☐ Indigenous methodology
- ☐ Participatory research
- ☐ Other qualitative study (non-specific, or write in ‘Other’)
- ☐ Mixed Method
- ☐ Health services research
- ☐ Health economics research
- ☐ Implementation research
- ☐ Policy research
- ☐ Other [ ]

6.2 (OPTIONAL) Further details / comments about the methods, methodology or study design

METHOD: technique to gather, analyze or report data. METHODOLOGY: principles /procedures / study designs.
If you were unsure about your previous answer, please explain here to help us with the final coding.

[ ]

6.3 List any conditions / diseases / TM diagnoses / participant characteristics

If not applicable or relevant enter: n/a

[ ]

6.4 Number of participants

If not applicable or relevant enter: n/a

[ ]

6.5 Country/Regions where the study was conducted

Select all that apply

- ☐ No information reported
- ☐ Australia
- ☐ Brazil
- ☐ Canada
- ☐ China
- ☐ Germany
- ☐ India
- ☐ Iran
- ☐ Japan
- ☐ South Korea
- ☐ UK
- ☐ USA
- ☐ Other [ ]

6.8 (OPTIONAL) Further details about any METHODS or METHODOLOGIES, STUDY DESIGNS that were applied, discussed or recommended.

METHOD: technique to gather, analyze or report data. METHODOLOGY: principles /procedures / study designs.

[ ]

7 LITERATURE REVIEW / EVIDENCE SYNTHESIS

7.1 What type of review is this?

Specify in 'Other' if the review methodology is not listed.

- ☐ UNSURE how to classify (explain in 'Other')
- ☐ NARRATIVE review (i.e., not systematically conducted)
- ☐ SYSTEMATIC REVIEW of interventions (including synthesis without meta-analysis)
- ☐ SCOPING review (systematically conducted)
- ☐ REALIST review (systematically conducted)
- ☐ MIXED METHODS review (systematically conducted)
- ☐ INTERPRETIVE review (systematically conducted)
- ☐ BIBLIOMETRIC review / analysis (systematically conducted)
- ☐ NON-SPECIFIC systematically conducted review
- ☐ Other [ ]

7.2 (OPTIONAL) Further details/comments about the type of review and methods (including quality appraisal) used in the review.

If you were unsure about your previous answer, please explain here to help us with the final coding.

[ ]

7.3 What types of studies or evidence sources were included or reported?

- ☐ No information reported
- ☐ UNSURE how to classify (please write details in Comments section)
- ☐ Basic sciences / Preclinical (non-specific, or write in 'Other')
- ☐ In silico / computer simulations / AI
- ☐ In vivo / in vitro
- ☐ Network pharmacology
- ☐ System / Complexity Sciences
- ☐ Basic Experimental Studies Involving Humans (BESH)
- ☐ Randomized controlled trial (RCT)
- ☐ Non-randomized studies of interventions (NRSI)
- ☐ Case series / report
- ☐ N-of-1
- ☐ Other clinical research (non-specific, or write in 'Other')
- ☐ Longitudinal cohort study
- ☐ Cross sectional study
- ☐ Other quantitative methodology (non-specific, or write in 'Other')
- ☐ Anthropology / ethnomedicine
- ☐ Ethnopharmacology / ethnobotany
- ☐ Indigenous methodology
- ☐ Participatory research
- ☐ Other qualitative methodology (non-specific, or write in 'Other')
- ☐ Mixed Method
- ☐ Health services research
- ☐ Health economics research
- ☐ Implementation research
- ☐ Policy research
- ☐ Literature reviews
- ☐ History / Ancient text review
- ☐ Other

7.4 (OPTIONAL) Further details about any specific METHODS or METHODOLOGIES, STUDY DESIGNS that were reported, discussed or recommended in the review.

METHOD: technique to gather, analyze or report data. METHODOLOGY: principles /procedures / study designs.
If you were unsure about your previous answer, please explain here to help us with the final coding.

7.5 List any conditions / diseases / TM diagnoses

If not applicable or relevant enter: n/a

7.6 Number of studies

If not applicable or relevant enter: n/a

7.7 Country/Regions of the studies included in this review

Select all that apply

- ☐ No information reported
- ☐ Australia
- ☐ Brazil
- ☐ Canada
- ☐ China
- ☐ Germany
- ☐ India
- ☐ Iran
- ☐ Japan
- ☐ South Korea
- ☐ Thailand
- ☐ UK
- ☐ USA
- ☐ Other

8 OTHER EVIDENCE SOURCES

8.1 What methodologies were discussed or recommended?

METHODOLOGY: principles /procedures / study designs.
Select any that apply

- ☐ None
- ☐ UNSURE how to classify (write details in Comments section)
- ☐ Basic sciences / Preclinical (non-specific, or write in ‘Other’)
- ☐ In silico / computer simulations / AI
- ☐ In vivo / in vitro
- ☐ Network pharmacology
- ☐ System / Complexity Sciences
- ☐ Basic Experimental Studies Involving Humans (BESH)
- ☐ Randomized controlled trial (RCT)
- ☐ Non-randomized studies of interventions (NRSI)
- ☐ Case series / report
- ☐ N-of-1
- ☐ Other Clinical research (non-specific, or write in ‘Other’)

- ☐ Cross sectional study
- ☐ Other quantitative methodology (non-specific, or write in ‘Other’)
- ☐ Anthropology / ethnomedicine
- ☐ Ethnopharmacology / ethnobotany
- ☐ Indigenous methodology
- ☐ Participatory research
- ☐ Other qualitative methodology (non-specific, or write in ‘Other’)
- ☐ Mixed Method
- ☐ Health services research
- ☐ Health economics research
- ☐ Implementation research
- ☐ Policy research / documents
- ☐ Systematic review of interventions
- ☐ Other evidence synthesis (specify in 'Other')
- ☐ Historical / Ancient texts
- ☐ Other ______

8.2 (OPTIONAL) Further details about any specific METHODS or METHODOLOGIES, STUDY DESIGNS that were discussed or recommended

METHOD: technique to gather, analyze or report data. METHODOLOGY: principles /procedures / study designs.
If you were unsure about your previous answer, please explain here to help us with the final coding.

9 RESEARCH FRAMEWORKS, GUIDELINES & STRATEGIES

9.1 (OPTIONAL) If relevant describe the research framework, guidelines or strategy that was recommended or discussed.

Summarize or copy quotes “each within individual quotations marks”

10 PARADIGM ALIGNMENT

10.1 (OPTIONAL) Paradigmatic alignment between the TM being studied and the research approach(es) applied, recommended or discussed.

Research Approach = method, methodology, framework, guideline, strategy.
ONLY ANSWER if this is very relevant.
OTHERWISE select N/A

- ☐ PARADIGM SPECIFIC (an ancient or modern research approach that originates from the TM paradigm e.g., Indigenous methodology for studying Indigenous medicine)

- ☐ PARADIGM INCOMPATIBLE (incompatible use of a research approach from another paradigm, either biomedical or other TM paradigm e.g., trying to conduct a double-blind RCT when blinding is not possible)
- ☐ PARADIGM COMPATIBLE (appropriate use of a research approach from another paradigm e.g., qualitative research, real-world data, pragmatic trial, double-blind RCT of a herbal medicine with a placebo control)
- ☐ PARADIGM ADAPTED (adapting a research approach from another paradigm e.g., dual TM and biomedical diagnosis for the inclusion criteria or outcome measure for a clinical trial; combing participatory action research + Indigenous methodology for health services research)
- ☐ PARADIGM PLURALISTIC (co-existence of 2 or more incompatible approaches used side-by-side e.g., traditional knowledge + biomedical research are both used for decision-making, mixed method studies)
- ☐ Very relevant, but UNSURE what to select
- ☐ UNSURE if it is very relevant
- ☐ N/A not relevant

10.2 (OPTIONAL) Comments about paradigm alignment, including if unsure

Please provide further details, OR if you were unsure about your previous answer, please explain here to help us with the final coding.

11 OTHER COMMENTS

11.1 (OPTIONAL) Please provide any other relevant information here:

Summarize or copy quotes “each within individual quotations marks”

12 BASIC SCIENCE / PRECLINICAL

12.1 (OPTIONAL) Which of the following were applied, summarized, discussed or recommended for basic science or preclinical research?

Select all that apply

- ☐ n/a
- ☐ Safety / toxicity
- ☐ Quality control
- ☐ in silico

- ☐ Herbal complexity
- ☐ Mechanistic
- ☐ Translational research
- ☐ Novel approaches
- ☐ Research strategies
- ☐ Other [ ]

2. 12.2 (OPTIONAL) Please provide any other relevant information here:

Summarize or copy quotes “each within individual quotations marks”

[ ]

13 CITATION TRACKING

Please look at the reference list for relevant article to track

13.1 Are there any relevant citations in the bibliography?

These will be added to Title/Abstract screening.

- ☐ No
- ☐ Yes
- ☐ Did not check (apologies)

13.2 Copy any potentially relevant citation details below

please do the full bibliography search NOW, not later :-)

[ ]

13.3 Do you recommend other citation tracking?

List details below

- ☐ No
- ☐ Author tracking
- ☐ Project / study tracking
- ☐ Forward citation tracking
- ☐ Other [ ]

13.4 (OPTIONAL) details or comments about citation tracking

[ ]
