# Supplementary material for: Protocol for a scoping review of traditional medicine research methods, methodologies, frameworks and strategies
Source: Front Med (Lausanne). 2024 Jul 10;11:1409392. doi: 10.3389/fmed.2024.1409392 (PMC11267516; doi:10.3389/fmed.2024.1409392)
Supplement: Supplementary file 4 [file Data_Sheet_4.pdf]

Supplementary Material 4

Protocol for an Evidence Review of Traditional Medicine Research Methods, Methodologies, Frameworks and Strategies.

Nadine Ijaz*†, Jennifer Hunter†, Suzanne Grant, Kate Templeman

* Correspondence: Nadine.Ijaz@Carleton.ca

†These authors contributed equally to this work and share first authorship

4 Examples of draft charting tables

Table 1. Characteristics of included documents

	Count
**Total number of primary documents***	
**Reason(s) for inclusion**	
Summarized TM research approaches	
Critiqued or outlined the rationale for a TM research approach	
Provided formal recommendations for how to conduct/report TM research	
Applied a paradigmatically aligned research approach	
**Document type**	
Primary study / research (published in a peer review journal) †	
Secondary study / literature review (published in a peer review journal) †	
Other peer reviewed journal article e.g., editorial, commentary, critical analysis, hypothesis, theory, report †	
Formal guidelines (published in a peer review journal or grey literature)	
Letter to editor / response letter (published in a peer review journal	
Grey literature: research thesis / dissertation	
Grey literature: Other	

Book or book chapter	
Website	
**Publication year**	
1990 - 1999	
2000 - 2009	
2010 - 2019	
2020 - 2024	
**WHO region for all authors**	
African Region (AFR)	
Region of the Americas (AMR)	
South-East Asian Region (SEAR)	
European Region (EUR)	
Eastern Mediterranean Region (EMR)	
Western Pacific Region (WPR)	
**Publication language †**	
English	
Chinese	
Korean	
Japanese	
Thai	
Portuguese	
French	
Spanish	
(NB – this may be expanded depending on results)	
Other:	
**Declarations**	
Funding & interests declared	
Interests declared; funding not declared	
Funding declared; interests not declared	
Published by an organization	

Not declared	

* some studies and research programs were published in > 1 document; † some primary documents were published in > 1 language

Table 2. Traditional medicine that was evaluated, studied or discussed*

**TM type**	**Count**
**Any TM (i.e., general / not specific)**	
**TM whole systems**	
Any TM whole system (i.e., not specific)	
Indigenous medicine *(NB – this may be expanded depending on results)*	
Anthroposophical medicine	
Ayurvedic Medicine	
Chiropractic medicine	
Homeopathic medicine	
Naturopathic medicine	
Osteopathic medicine	
Persian medicine	
Siddha medicine	
Traditional East Asian Medicine (e.g., Chinese Medicine, Kampo, Korean Medicine etc.)	
Unani medicine	
(NB – this may be expanded depending on results)	
Other:	
**Modalities**	
Any TM modality (i.e., not specific)	
Acupuncture and other acupoint therapies	
Arts-based / expressive therapies	
Tai Chi / Qigong	
Yoga	

Manual / touch therapies *(NB – this might be expanded depending on results)*	
Meditation	
(NB – this may be expanded depending on results)	
Other:	
**TM products and foods**	
Any TM product (i.e., not specific)	
Botanicals / herbal medicine	
Nutraceuticals (not herbs)	
Medicinal foods	
(NB – this may be expanded depending on results)	
Other:	
**Other**	
Integrative Medicine	
(NB – this may be expanded depending on results)	

* counts are from both primary (n =) and secondary documents (n =)

Table 3. Traditional medicine research approaches applied of discussed *

**Research approaches**	**Count**
Method	
Methodology	
Framework	
Guideline	
Strategy	
**Research domains**	
Basic sciences / Preclinical	
Clinical research	
Health Services Research	
Economic Evaluations	
Epidemiology	

Social Sciences	
Policy Research	
Ethnomedicine (e.g., Ethnobotany / Ethnopharmacology / Medical Anthropology)	
(NB – this may be expanded depending on results)	
Other:	
**Purpose of the research approach**	
Develop or apply TM-specific research methods or methodologies	
Evaluate TM system or intervention	
Further understand or develop the TM therapeutic paradigm	
Develop or apply TM diagnostic criteria / outcome measurement	
Inform decision making (e.g., CPGs, policy)	
Collect information about TM use / practitioners / services	
Improve research conduct / reporting (e.g., CONSORT or other guidelines)	
(NB – this may be expanded depending on results)	
Other:	

* counts are from both primary (n =) and secondary documents (n =)
